# PD-1/PD-L1 axis in organ fibrosis

**DOI:** 10.3389/fimmu.2023.1145682

**Published:** 2023-05-19

**Authors:** Youliang Zhao, Yaqian Qu, Changfu Hao, Wu Yao

**Affiliations:** Department of Occupational Health and Occupational Disease, College of Public Health, Zhengzhou University, Zhengzhou, Henan, China

**Keywords:** PD-1, PD-L1, immune checkpoint, fibrosis, immunomodulatory

## Abstract

Fibrosis is a pathological tissue repair activity in which many myofibroblasts are activated and extracellular matrix are excessively accumulated, leading to the formation of permanent scars and finally organ failure. A variety of organs, including the lung, liver, kidney, heart, and skin, can undergo fibrosis under the stimulation of various exogenous or endogenous pathogenic factors. At present, the pathogenesis of fibrosis is still not fully elucidated, but it is known that the immune system plays a key role in the initiation and progression of fibrosis. Immune checkpoint molecules are key regulators to maintain immune tolerance and homeostasis, among which the programmed cell death protein 1/programmed death ligand 1 (PD-1/PD-L1) axis has attracted much attention. The exciting achievements of tumor immunotherapy targeting PD-1/PD-L1 provide new insights into its use as a therapeutic target for other diseases. In recent years, the role of PD-1/PD-L1 axis in fibrosis has been preliminarily explored, further confirming the close relationship among PD-1/PD-L1 signaling, immune regulation, and fibrosis. This review discusses the structure, expression, function, and regulatory mechanism of PD-1 and PD-L1, and summarizes the research progress of PD-1/PD-L1 signaling in fibrotic diseases.

## 1 Introduction

Fibrosis is a pathological process in which fibrous connective tissue (mainly collagen) accumulates excessively around inflamed or damaged tissues to form permanent scar structures, affecting organ function and eventually leading to death (1). Collagen deposition in fibrosis is necessary for biological activities such as wound healing and tissue repair. Under physiological conditions, collagen deposition is finely regulated by a complex molecular network to ensure the effective operation of tissue repair. When the tissue damage is too severe or occurs continuously and repeatedly, the tissue repair program is disturbed and causes a progressive irreversible fibrotic response, which eventually leads to organ fibrosis (2). Thus, fibrosis is an end-stage manifestation of an uncontrolled pathological tissue repair response. Studies have shown that fibrosis occurs in almost all types of tissues in the human body (3). Although the fibrotic response of different organs may show phased adaptive changes under different pathological conditions, long-term tissue damage will eventually lead to substantial scar formation and fibrotic lesions, which further suggests that fibrosis is closely related to human diseases (4). The incidence of fibrosis diseases in various organs is increasing, but the specific therapeutic strategies targeting the fibrogenesis process are particularly lacking. Therefore, it is of great significance to further clarify the molecular mechanism of different types of fibrotic diseases, reveal the general rules of fibrotic response, and screen specific targets for improving the ability to cure fibrotic diseases.

Programmed cell death protein 1 (PD-1) and programmed death ligand 1 (PD-L1) are the key immunosuppressive signals to maintain central and peripheral immune tolerance, inhibit autoimmune responses, and regulate immune homeostasis (5). PD-1/PD-L1 axis plays an important role in T cell response, which can inhibit T cell activation, proliferation, cytokine secretion, induce T cell apoptosis, and exhaustion, thereby inhibiting T cell immune effector function (6, 7). Given this, the PD-1/PD-L1 axis has been widely concerned in a variety of tumors. The expression of PD-L1 on the surface of tumor cells is generally up-regulated, which binds to PD-1 on the surface of T cells to inhibit the anti-tumor immunity of T cells, thereby achieving immune escape (8). Based on this, immune checkpoint inhibitors targeting PD-1/PD-L1 signaling have made remarkable achievements in the clinical treatment of a variety of tumors, which has significantly improved the ability of tumor immunotherapy. The antitumor mechanism of PD-1/PD-L1 inhibitors is to limit immunosuppressive signals, restore cytotoxic T-cell function, and achieve tumor clearance (9). The success of PD-1/PD-L1 blockade therapy in tumors also provides certain enlightenment to the basic and clinical research of other immune-related diseases. It has been found that PD-1/PD-L1 axis is involved in the occurrence and development of autoimmune diseases, chronic infection, sepsis, cardiovascular diseases, neurodegenerative diseases, and fibrosis diseases (6, 7, 10). With the deepening of PD-1/PD-L1 research, in addition to T lymphocytes, more and more immune cell functions have been confirmed to be regulated by PD-1/PD-L1 signaling, including monocytes, dendritic cells, natural killer cells, and B cells (11, 12). It can be seen that PD-1/PD-L1 signaling pathway plays a crucial role in maintaining immune homeostasis and is closely related to human diseases. In recent years, the role of PD-1/PD-L1 axis in organ fibrosis has also been confirmed. Given the close relationship between the PD-1/PD-L1 axis, immune regulation, and fibrosis, in this review, we discuss the structure, expression, function, and regulatory mechanism of PD-1 and PD-L1, and summarize the research of PD-1/PD-L1 axis in fibrotic diseases of lung, liver and other organs. We hope to provide new ideas for further understanding the immunological mechanism of fibrosis and constructing PD-1/PD-L1-based immunotherapy strategies for fibrotic disease.

## 2 The pathogenesis of fibrosis

**Figure 1:**
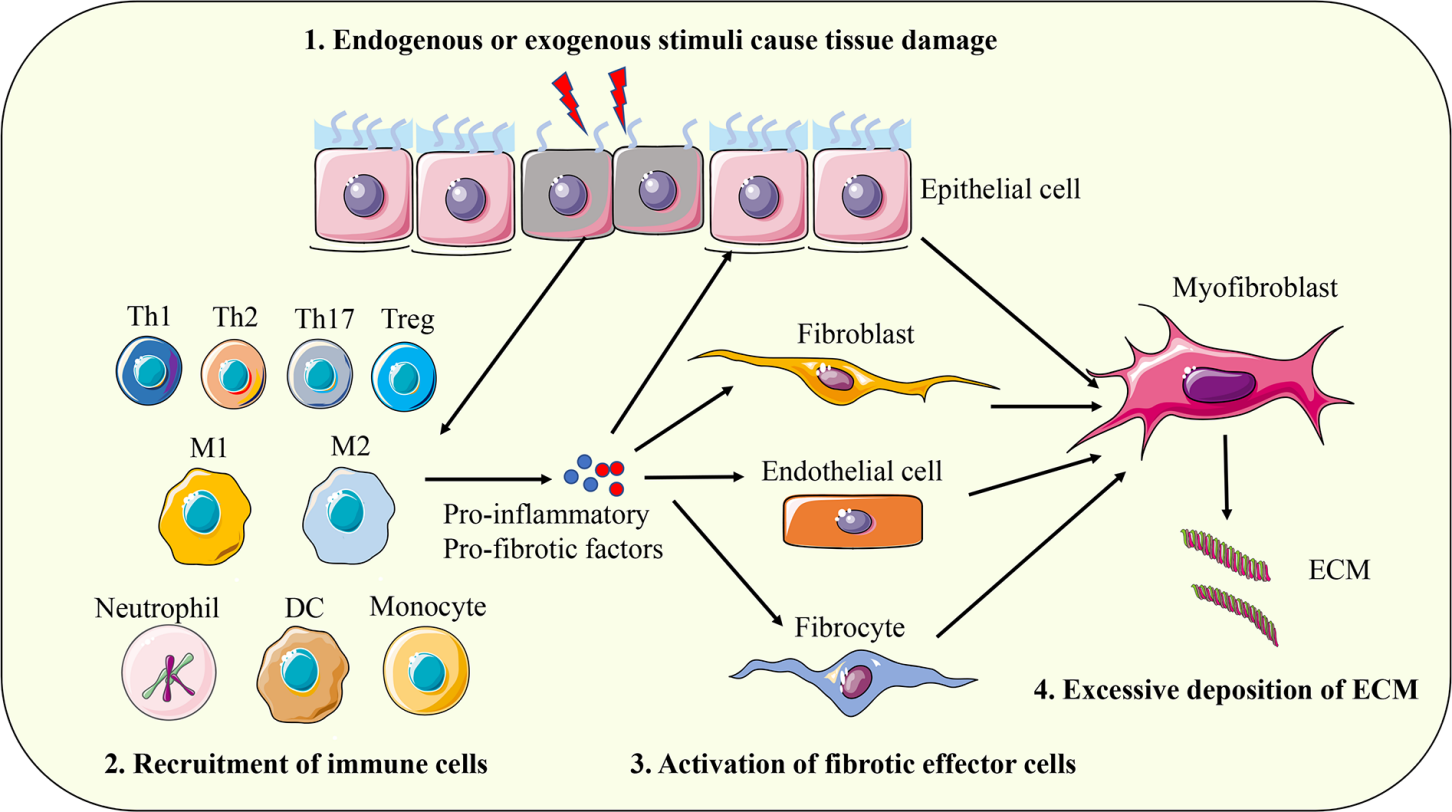
The pathogenesis of fibrosis. First, tissue injury caused by endogenous or exogenous factors initiates the inflammatory response; Second, immune cells from peripheral blood and peripheral lymphoid tissue are massively recruited to the injured site. Third, activation of key fibrotic effector cells, myofibroblasts; Fourth, the excessive production and deposition of extracellular matrix components eventually leads to organ failure. Th, T helper cell; Treg, regulatory T cell; M1, classically activated macrophages; M2, alternative activated macrophages; DC, dendritic cell; ECM, extracellular matrix.

A variety of endogenous and exogenous factors can initiate the body’s fibrotic response, such as genetic factors, autoimmune inflammation, chronic infection, repeated exogenous toxic exposure, obesity, hypertension, and diabetes (13). Although the pathogenic factors of fibrosis are different in different organs and different disease backgrounds, the fibrosis process can be divided into four main stages based on pathological characteristics. First, tissue injury caused by endogenous or exogenous factors initiates the inflammatory response; Second, immune cells from peripheral blood and peripheral lymphoid tissue are massively recruited to the injured site. Third, activation of key fibrotic effector cells, myofibroblasts; Fourth, the excessive production and deposition of extracellular matrix components eventually lead to organ failure (4). The above series of physiological and pathological processes are comprehensive manifestations of the interaction of a variety of cells, molecules, and signaling pathways (**Figure 1**). The pathological response to fibrosis and the physiological response to tissue repair have similar biological mechanisms in essence. When the tissue damage signal cannot be eliminated in time, the protective tissue repair process gradually changes to the harmful fibrogenesis process (14). The cells involved in the fibrotic response mainly include inflammatory cells (such as macrophages, monocytes, neutrophils, and T lymphocytes), epithelial cells, endothelial cells, and fibroblasts (4, 15, 16). Myofibroblasts are the key effector cells that mediate fibrotic responses. They can secrete large amounts of ECM components, possess enhanced proliferation, migration, and contraction abilities, and are resistant to apoptosis (17). Fibroblasts are the major precursors of myofibroblasts, which can be activated and transdifferentiated into myofibroblasts by pro-fibrotic factors such as transforming growth factor β1 (TGF-β1). In addition, epithelial cells, endothelial cells, pericytes, fibrocytes, and even macrophages can convert into myofibroblasts under certain conditions and participate in the fibrotic response (18, 19). With the progress of fibrosis, myofibroblasts produce and release excessive ECM, which leads to scar formation and affects tissue structure. The contractile characteristics of myofibroblasts also cause deformation of tissue parenchyma and eventually lead to structural and functional abnormalities of tissues and organs. The main components of ECM proteins in fibrotic scar are type I and type III collagen, fibronectin, and laminin, which are highly conserved in different tissues (4). ECM not only affects tissue structure and cell interaction, but also directly regulates cell proliferation, differentiation, metabolism, and migration (20, 21). In summary, myofibroblasts and their secreted ECM components play a key role in the fibrotic response. Based on this, a large number of studies have focused on elucidating the molecular mechanism of transdifferentiation of various cells into myofibroblasts, and TGF-β1-mediated signaling pathway has been proven to be a key driver of fibrosis response (22). In addition, a variety of cytokines such as interleukin 4 (IL-4), IL-6, IL-13; Chemokines such as monocyte chemoattractant protein-1 (MCP-1); Growth factors such as vascular endothelial growth factor (VEGF) and platelet-derived growth factor (PDGF); And other mediators such as reactive oxygen species (ROS) and lipids are involved in the regulation of fibrosis (16, 23).

## 3 Immunological regulation of fibrosis

The immune system plays a key regulatory role in various fibrotic diseases. Both acute and persistent chronic inflammation can initiate fibrosis, and the occurrence of inflammation involves a variety of innate and adaptive immune cells (16, 24). Under inflammation conditions, damaged cells can release a series of chemokines to recruit circulating monocytes and neutrophils to the site of tissue injury, where monocytes can further differentiate into macrophages to remove foreign microorganisms, particles, damaged cells, and cell debris, thereby promoting tissue repair and maintaining immune homeostasis. In the process of removing the risk factors that cause tissue damage, macrophages and neutrophils are also accompanied by the release of a series of active substances that are toxic to normal cells, such as reactive oxygen species, reactive nitrogen species, and pro-inflammatory cytokines. Therefore, when the risk factors are eliminated and these immune cells and cytokines cannot be reduced in time, it will lead to further deterioration of tissue damage, thus activating the fibrosis response (2, 14, 25). The high plasticity of macrophages enables them to acquire unique phenotypes and functions in different environments. Classically activated macrophages (M1) are mainly induced by proinflammatory cytokines such as interferon-γ (IFN-γ) and mediate inflammatory and anti-tumor immune responses. Alternatively activated macrophages (M2) are mainly induced by Th2 cytokines such as IL-4 and participate in immunosuppressive and profibrotic responses (26). In addition, DC, eosinophils, basophils, and mast cells have also been confirmed to participate in liver, lung, and kidney fibrosis (2). In addition to the innate immune cells mentioned above, the role of adaptive immune T lymphocytes in fibrosis has also been extensively studied. Existing evidence shows that T helper 1 (Th1) cells, Th2, Th17, and regulatory T cell (Treg) can play opposite roles in promoting or inhibiting fibrosis progression in different organs and different immune microenvironments (16, 27). Th1 cytokine IFN-γ can not only inhibit the differentiation of Th2 cells but also block the pro-fibrotic signal mediated by TGF-β1, thus exerting a significant anti-fibrotic effect (28, 29). However, another important Th1 cytokine tumor necrosis factor α (TNF-α) has been shown to have pro-fibrotic effects in a variety of animal models (30, 31). As for Th2, they are generally considered to release IL-4, IL-5, and IL-13, which have pro-fibrotic effects. For example, IL-13 can not only induce the expression and activation of key pro-fibrosis factor TGF-β1 but also directly promote the activation of fibroblasts (32, 33). However, in the acute tubular cell necrosis model, IL-4 can inhibit renal tubulointerstitial fibrosis by promoting tissue regeneration (34). IL-17A released by Th17 cells can up-regulate the expression of TGF-β1 and directly act on fibroblasts to promote their transformation into myofibroblasts (35). However, low dose of IL-17A could prevent and reverse diabetic renal fibrosis (36). Treg cells secrete two important cytokines, IL-10 and TGF-β1, which also play a complex role in fibrosis. TGF-β1 is a potent pro-fibrotic factor that can induce the transdifferentiation of fibroblasts, epithelial cells, and endothelial cells into myofibroblasts. Therefore, Treg can secrete large amounts of TGF-β1 to mediate the pro-fibrotic response (37). IL-10 is an important immunosuppressive factor, which can reduce tissue damage by inhibiting inflammatory response, thus playing an anti-fibrosis role (38). Therefore, T cell-mediated immune regulation plays a pivotal role in fibrosis, and its effector function is closely related to the state of cell polarization and the characteristics of immune microenvironment. In summary, the fibrosis response is precisely regulated pathophysiological processes involving a variety of immune cells and corresponding mediators. In-depth exploration of the immunological regulation mechanism can provide theoretical basis for further elucidating the pathogenesis of fibrosis.

## 4 Basis of the PD-1/PD-L1 axis

### 4.1 Structure of PD-1 and PD-L1 protein

PD-1, also known as CD279, is encoded by the *PDCD1* gene located on human chromosome 2 (39). PD-1 is a type I transmembrane protein belonging to the CD28 immunoglobulin superfamily, which is composed of an IgV-like extracellular domain, a transmembrane domain, and a cytoplasmic domain. The cytoplasmic tail of PD-1 contains two key tyrosine signaling motifs, termed the immunoreceptor tyrosine inhibitory motif (ITIM) and the immunoreceptor tyrosine switch motif (ITSM), that mediate downstream signaling (40, 41).

PD-L1, also known as CD274 or B7-H1, is encoded by the *PDCDL1* gene located on human chromosome 9, which belongs to the B7 immunoglobulin superfamily (42). As the main ligand of the PD-1 receptor, PD-L1 is also a typical type I transmembrane protein, containing extracellular IgV-like and IgC-like domains, a hydrophobic transmembrane domain, and a cytosolic domain consisting of 30 amino acids. However, in contrast to PD-1, the cytoplasmic tail of PD-L1 does not contain classical signal transduction motifs (5, 43).

### 4.2 Expression of PD-1 and PD-L1

Under physiological conditions, the expression of PD-1 and PD-L1 is an important signal to maintain immune tolerance and homeostasis. Under pathological conditions such as inflammation, autoimmune response, and cancer, the expression of PD-1 and PD-L1 can be induced by various factors such as cytokines, growth factors, and gene mutations to participate in disease progression.

PD-1 is mainly expressed in a variety of immune cells, including activated T cells, B cells, NK cells, macrophages, DCs, and monocytes (5, 7). Among the T cell subtypes, exhausted T cells, Treg cells, follicular helper T cells, follicular regulatory T cells, and memory T cells were identified to express PD-1, and high expression of PD-1 was an important feature of exhausted T cells (6). PD-1 is also a hallmark of effector T cells because it is not expressed by resting T cells and is dramatically upregulated when T cell receptor (TCR) mediated T-cell activation signals are activated in response to antigenic peptides (44). Hence, the positive signal mediated by the binding of antigen peptides-MHC complex to TCR is the most important signal to induce PD-1 expression in T cells. PD-1 expression is rapidly downregulated when T-cell activation signals are abrogated, otherwise it remains at high levels (45). In addition to TCR signaling, cytokines such as IL-10 and TGF-β can also induce the expression of PD-1 (46, 47).

PD-L1 is widely expressed in a variety of hematopoietic cells such as T cells, B cells, macrophages, DC, monocytes, mesenchymal stem cells, non-hematopoietic cells such as epithelial cells, endothelial cells, fibroblasts, keratinocytes, and various of cancer cells (12, 30, 48). Multiple proinflammatory factors such as IFN-γ can induce PD-L1 expression, and thus PD-L1 expression is usually associated with persistent inflammatory responses (49). In tumor immunity, IFN-γ secreted by tumor-infiltrating T cells induces the up-regulation of PD-L1 expression in tumor cells, thereby promoting the formation of immunosuppressive microenvironment, which may be one of the key mechanisms of tumor adaptive resistance.

### 4.3 PD-1/PD-L1 interaction

**Figure 2:**
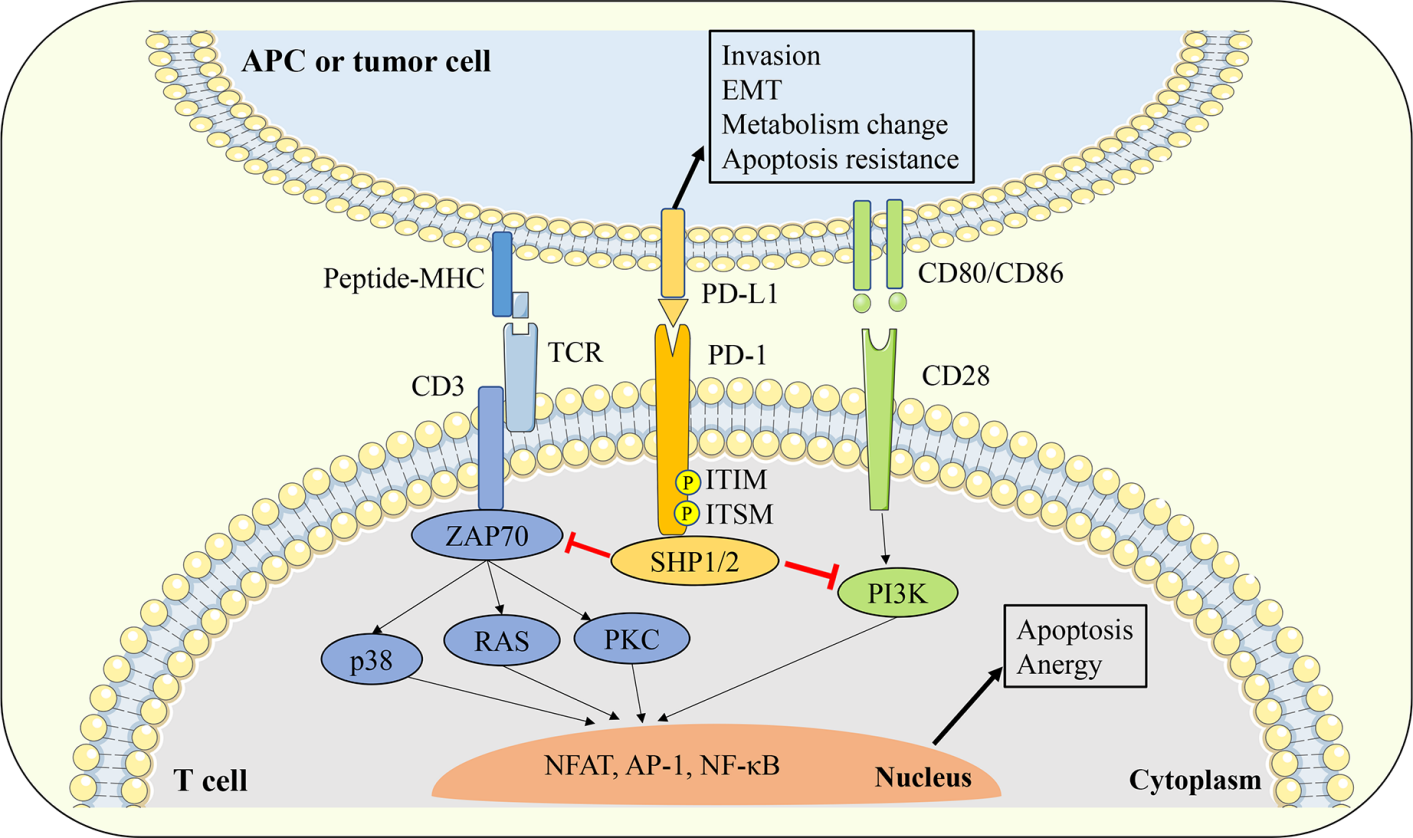
Interaction between PD-1 and PD-L1. The engagement of PD-1 receptor on the surface of T cells to PD-L1 ligands expressed on the surface of APCs or tumor cells will cause the conformational change of PD-1, leading to the phosphorylation of tyrosine in the ITIM and ITSM signal motifs in the intracellular domain of PD-1. Phosphorylated PD-1 recruits protein tyrosine phosphatases such as SHP1/2, and SHP1/2 could inhibit the downstream TCR and CD28 signal cascade. Thereby leads to T cell activation and proliferation inhibition, exhaustion, Treg cell differentiation, apoptosis initiation, cytokine production reduction, and metabolic changes. On the other hand, PD-L1 can also transmit biological signals to PD-L1 expressing cells and affect tumor cell invasion, EMT and apoptosis resistance. APC, antigen presentation cell; EMT, epithelial-mesenchymal transition; TCR, T cell receptor; ZAP70, zeta-chain-associated protein kinase; PKC, protein kinase C; SHP1/2, Src homology region 2 domain-containing phosphatase 1/2; ITIM, immunoreceptor tyrosine based inhibitory motif; ITSM, immunoreceptor tyrosine based switch motif;PI3K, phosphoinositide 3-kinase/serine-threonine kinase 1; NFAT, nuclear factor of activated T cells; AP-1, activator protein 1; NF-κB, nuclear factor kappa-light-chain-enhancer of activated B cells.

The engagement of the PD-1 receptor on the surface of T cells to PD-L1 ligands expressed on the surface of antigen presentation cells (APCs) or tumor cells will cause the conformational change of PD-1, leading to the phosphorylation of tyrosine in the ITIM and ITSM signal motifs in the intracellular domain of PD-1. Phosphorylated PD-1 recruits protein tyrosine phosphatases such as Src homology region 2 domain-containing phosphatase 1/2 (SHP1/2), and SHP1/2 could induce the dephosphorylation of TCR and CD28 downstream signal kinases such as Zeta-chain-associated protein kinase (ZAP70) and phosphoinositide 3-kinase/serine-threonine kinase 1 (PI3K/AKT). In turn, T cell activation signals mediated by ZAP70 and PKC such as protein kinase C (PKC), extracellular signal regulated kinase (ERK), phospholipase Cγ (PLCγ), and RAS will be blocked (**Figure 2**). Eventually, the above signaling cascade leads to T cell activation and proliferation inhibition, exhaustion, Treg cell differentiation, apoptosis initiation, cytokine production reduction, and metabolic changes (50–53). Some studies have shown that compared with TCR signaling, the PD-1 pathway may affect T cell activation mainly by interfering with CD28 costimulatory signaling (54).

Due to the lack of classical signaling motifs in the intracellular domain of PD-L1 protein, the study of PD-1/PD-L1 interaction usually focuses on the signal transduction process in PD-1 expressing cells, but less attention is paid to the intracellular signaling mediated by PD-L1. However, it has been shown that the engagement of PD-1 and PD-L1 can also transmit biological signals to PD-L1-expressing cells and affect their functions. For example, stimulation of tumor cells with PD-1 recombinant proteins can activate PD-L1 signaling and enhance the apoptosis resistance of tumor cells (55). In an *in vitro* model lacking T cells, blocking PD-L1 signaling in tumor cells with PD-L1 antibody significantly affected their glucose metabolic activity, thereby inhibiting cell viability (56).

### 4.4 Non-cytomembrane PD-1 and PD-L1

**Figure 3:**
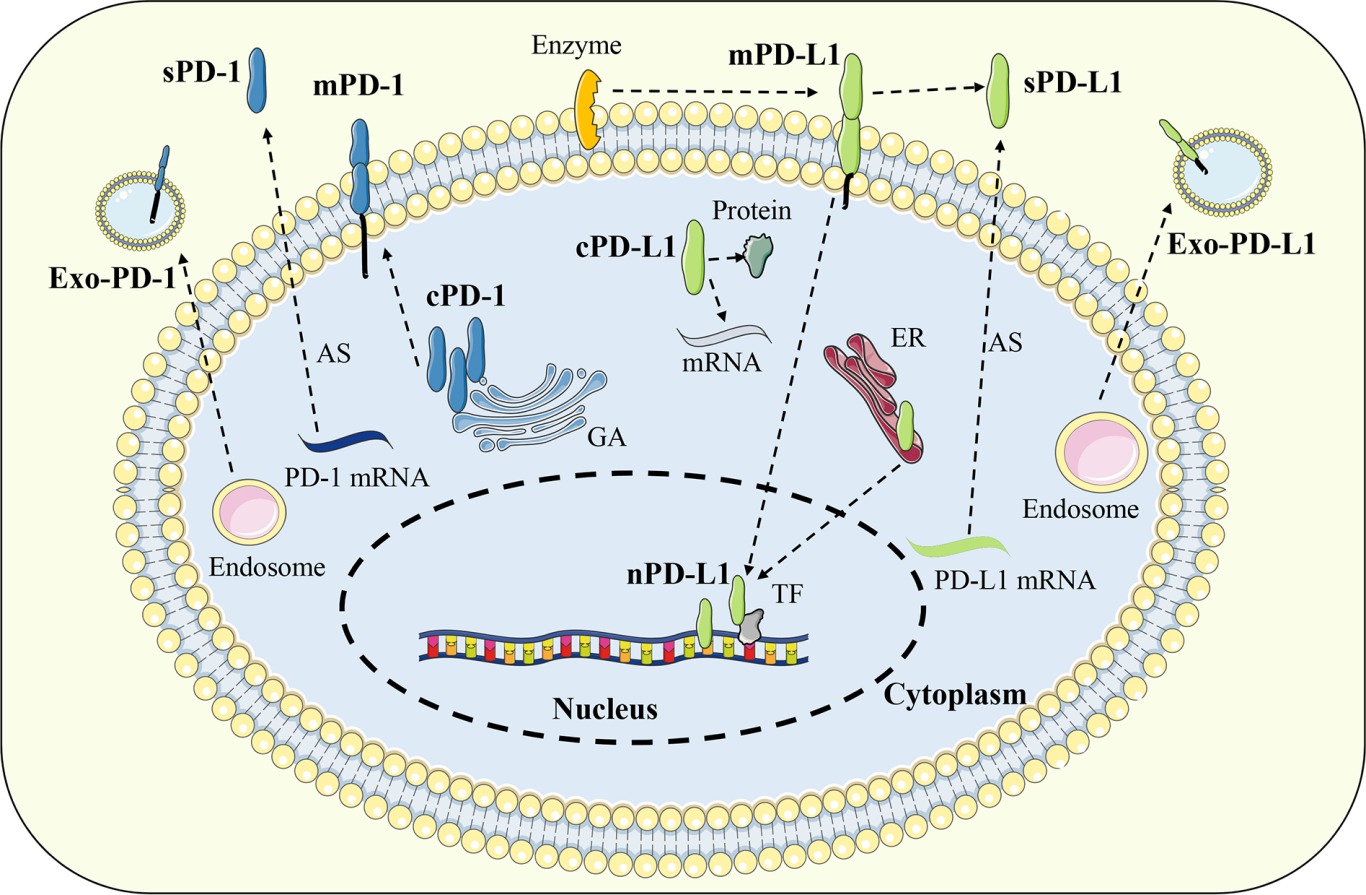
Distribution of PD-1 and PD-L1. In addition to the classical membrane-bound form, PD-1 and PD-L1 can also be distributed in extracellular or intracellular regions in a non-membrane-bound form. Extracellular PD-1 and PD-L1 are mainly present in soluble form or located on the surface of exosome. Intracellular PD-L1 can be distributed in the cytoplasm or nucleus while PD-1 was found only in the cytoplasm. Exo-PD-1/PD-L1, exosomal PD-1/PD-L1; sPD-1/PD-L1, soluble PD-1/PD-L1; mPD-1/PD-L1, membrane PD-1/PD-L1; cPD-1/PD-L1, cytoplasmic PD-1/PD-L1; nPD-L1, nuclear PD-L1; AS, alternative splicing; GA, golgi apparatus; ER, endoplasmic reticulum; TF, transcription factor.

In addition to the classical membrane-bound form, studies have confirmed that PD-1 and PD-L1 can also be distributed in extracellular or intracellular regions in a non-membrane-bound form (57). Extracellular PD-1 and PD-L1 are mainly present in various body fluids in soluble form or located on the surface of exosome membrane. Soluble PD-1 (sPD-1) or soluble PD-L1 (sPD-L1) is generated by alternative splicing of mRNA or membrane protein proteolysis, while exosomal PD-1 (Exo-PD-1) or exosomal PD-L1 (Exo-PD-L1) is released by the fusion of multivesicular bodies with the cell membrane. Intracellular PD-L1 can be distributed in the cytoplasm or nucleus and exert unique signal transduction functions depending on different mechanisms (58) (**Figure 3**).

#### 4.4.1 Extracellular PD-1 and PD-L1

Extracellular PD-1 and PD-L1 mainly include free soluble forms or exosomal membrane-bound forms, which can be distributed throughout the body with blood circulation. Studies have shown that some soluble and exosomal forms of PD-1 and PD-L1 maintain intact immune activity and can bind to their cell membrane receptors/ligands to exert immunomodulatory functions (59). In immune checkpoint blockade therapy, extracellular PD-1 and PD-L1 can act as molecular decoys to specifically bind to therapeutic antibodies, thereby affecting efficacy (60). In addition, due to the wide distribution and easy detection of extracellular soluble and exosomal PD-1 and PD-L1, they have great value as biomarkers for disease diagnosis or prognosis evaluation (57).

sPD-1 and sPD-L1 are generated by alternative splicing of mRNA during transcription or proteolysis of cell membrane PD-1 and PD-L1, lacking typical transmembrane domains (61, 62). The *PDCD1* gene contains five exons, which are responsible for encoding the extracellular, transmembrane, and intracellular structures of the PD-1 protein. Among them, the transmembrane domain of PD-1 is encoded by exon 3, and its selective deletion leads to the production of sPD-1. When only exon 3 is missing, sPD-1 has an intact extracellular domain and can specifically bind to PD-L1 ligand (5, 63). For PD-L1, the amplification of the endogenous retroelement LINE-2A in the intron of the PDCDL1 gene will lead to the deletion of the transmembrane and signaling domains of PD-L1 protein, thereby inducing the production of sPD-L1. Moreover, this type of sPD-L1 cannot activate the PD-1 signal but instead acts as a molecular decoy to antagonize the immunosuppressive activity of membrane PD-L1 (64). Other studies have reported that a variety of endogenous metalloproteinases are involved in the proteolysis of membrane PD-L1, resulting in the production of sPD-L1. For example, matrix metalloproteinase 9 (MMP9) and MMP13 catalyze the degradation of PD-L1 and PD-L2 on the surface of fibroblasts, inducing the production of sPD-L1 and reducing the ability of fibroblasts to activate the PD-1 signaling pathway, thereby indirectly promote T cell immunity (65). Thus, the biological activity that sPD-1 and sPD-L1 possess depends on how it is generated. When they contain an intact extracellular domain, they can specifically bind to the corresponding receptor or ligand to mediate immune regulation. Among them, the function of sPD-L1 is similar to that of membrane-bound PD-L1, which activates inhibitory immune signals by binding to PD-1 (59). sPD-L1 can be released by a variety of PD-L1-positive tumor cells to inhibit the effector function of T cells. Combined with the finding that sPD-L1 levels are significantly increased in the serum of cancer patients, it is suggested that sPD-L1 may be an important mediator for tumor cells to achieve immune escape (60, 66). Compared with PD-L1-mediated immunosuppressive effect, sPD-1 can prevent the activation of PD-1 signal by competitive binding to PD-L1, and indirectly enhance the anti-tumor immune effect (67). In melanoma, it has been found that intravenous injection of a plasmid encoding the extracellular domain of PD-1 can block the PD-1/PD-L1 signaling pathway and inhibit the lung metastasis and drug resistance of tumor cells (68). In conclusion, sPD-1 and sPD-L1 have a wide range of sources and diverse functions, and their immunomodulatory mechanism and clinical application value worth to be further exploring.

Exo-PD-1 and Exo-PD-L1 are localized on the exosome membrane, maintain the same protein structure as their cell membrane form, and are secreted to the outside of the cell with the release of exosomes (69). Many tumor cells, including lung cancer, prostate cancer, breast cancer, and melanoma, can release PD-L1-expressing exosomes, which prevent the activation of effector T cells by binding to PD-1 and promote the formation of a suppressive tumor immune microenvironment (58). Inhibition of genes related to exosome synthesis and release, such as N-SMase2 and Rab27a, can reduce the level of exosome PD-L1 by blocking the release of exosomes, and significantly improve the efficacy of anti-PD-L1 immunotherapy (70). The level of Exo-PD-L1 can be significantly upregulated in response to the stimulation of inflammatory factors such as IFN-γ (69). Therefore, it can be used to evaluate the response rate and efficacy of immune checkpoint inhibitor therapy. The high level of Exo-PD-L1 in the peripheral blood of patients before treatment may reflect the severe exhaustion of T cells, suggesting that it is difficult to ensure the efficacy of immune checkpoint inhibitors. If the level of Exo-PD-L1 in the peripheral blood of patients increases after treatment, it often indicates the recovery of T cell immune response and the restart of anti-tumor immunity (71, 72). Compared with sPD-L1, Exo-PD-L1 can be co-expressed with MHC molecules and thus has a more significant immunosuppressive ability (73). Due to the stability of exosomes, Exo-PD-L1 can not only regulate the local immune microenvironment but also enter the blood circulation to coordinate systemic immune homeostasis (74). In contrast to PD-L1, Exo-PD-1 was identified only in T cell-derived exosomes. Activated T cells can release exosomes carrying PD-1, which can bind to the cell surface or Exo-PD-L1 as a molecule decoy and induce PD-L1 internalization through clathrin-mediated endocytosis, thereby preventing the activation of PD-1 signaling and indirectly inhibiting PD-L1-mediated immunosuppression (75). In addition, T cell-derived Exo-PD-1 may be used to evaluate the clinical response rate of metastatic melanoma to ipilimumab treatment (76). In conclusion, Exo-PD-1 and Exo-PD-L1 can not only be used as therapeutic targets but also be applied to evaluate the clinical response rate and efficacy of immune checkpoint blockade.

#### 4.4.2 Intracellular PD-1 and PD-L1

Intracellular PD-L1 could be located in the cytoplasm or nucleus. Nuclear PD-L1 (nPD-L1) can regulate the transcription procedure by directly binding to DNA or transcription factors, and then affect cell activities (77). Currently, nPD-L1 expression has been found in many types of tumor cells. In non-small cell lung cancer, nPD-L1 can directly bind to the transcription factor SP1 and upregulate the expression of growth arrest-specific 6 (GAS6). GAS6 promotes tumor cell proliferation through the downstream MerTK signaling pathway (78). In addition, nPD-L1 can also activate inflammatory pathways and promote the presentation of tumor antigens, which may be related to the high response rate of PD-1/PD-L1 blockade in tumors with high levels of PD-L1. At present, the formation mechanism of nPD-L1 is not clear. At first, it was thought to originate from the endocytosis of membrane PD-L1. However, subsequent studies have found that nPD-L1 contains more complex glycosylation groups than membrane PD-L1, suggesting that part of PD-L1 may undergo highly glycosylated reactions after translation in the endoplasmic reticulum and Golgi apparatus and then be directly transported to the nucleus (79). Cytoplasmic PD-L1 (cPD-L1) has also been confirmed to exist in tumor cells such as the lung, kidney, and pancreas, as well as non-tumor cells such as macrophages, fibroblasts, and epithelial cells (80). In tumor cells, PD-L1 can rely on signal transducer and activator of transcription 3 (STAT3) signaling pathway to accumulate in the cytoplasm. Furthermore, cPD-L1 affects the anti-tumor effect of resveratrol by preventing the nuclear translocation of cytochrome c oxidase subunit II (COX2) (81, 82). In addition, similar to the DNA-binding properties of nPD-L1, cPD-L1 can also bind to mRNA to maintain its stability and facilitate the translation program. For example, Tu et al. found that cPD-L1 can act as an RNA-binding protein to regulate the mRNA stability of *NBS1* and *BRCA1*, which are associated with DNA damage and radiation resistance in some tumor cells (83). Thus, in contrast to classical immunosuppressive signals mediated by membrane PD-L1, intracellular PD-L1 can directly participate in transcriptional or translational processes and regulate tumor cell survival.

Compared with PD-L1, there is a lack of research on intracellular PD-1, and only a few pieces of research on T cells have confirmed the existence of intracellular PD-1. Studies have shown that the localization of PD-1 in T cells changes with their phenotypic switching. Among them, PD-1 expression was upregulated and distributed on the cell surface during T cell activation, while the expression of PD-1 in resting Treg cells was mainly localized intracellularly. When the TCR signal of Treg cells was activated, intracellular PD-1 could translocate to the cell membrane, thereby mediating immune regulation (84). In addition, in activated T cells, PD-1 is also distributed near the Golgi apparatus, but not in endosomes and lysosomes, which may be related to the intracellular trafficking of PD-1 synthesis (85). In the peripheral blood of patients with advanced melanoma, PD-1 accumulates in the cytoplasm of circulating T lymphocytes, which can supplement the loss of PD-1 on T cell membrane and mediate tumor immune escape by affecting the T cell function (86). Taken together, the main function of intracellular PD-1 may be as a reservoir of cell membrane PD-1 that compensates for the upregulation of cell membrane PD-1 levels under specific circumstances.

## 5 Regulation of PD-1 and PD-L1 expression

**Figure 4:**
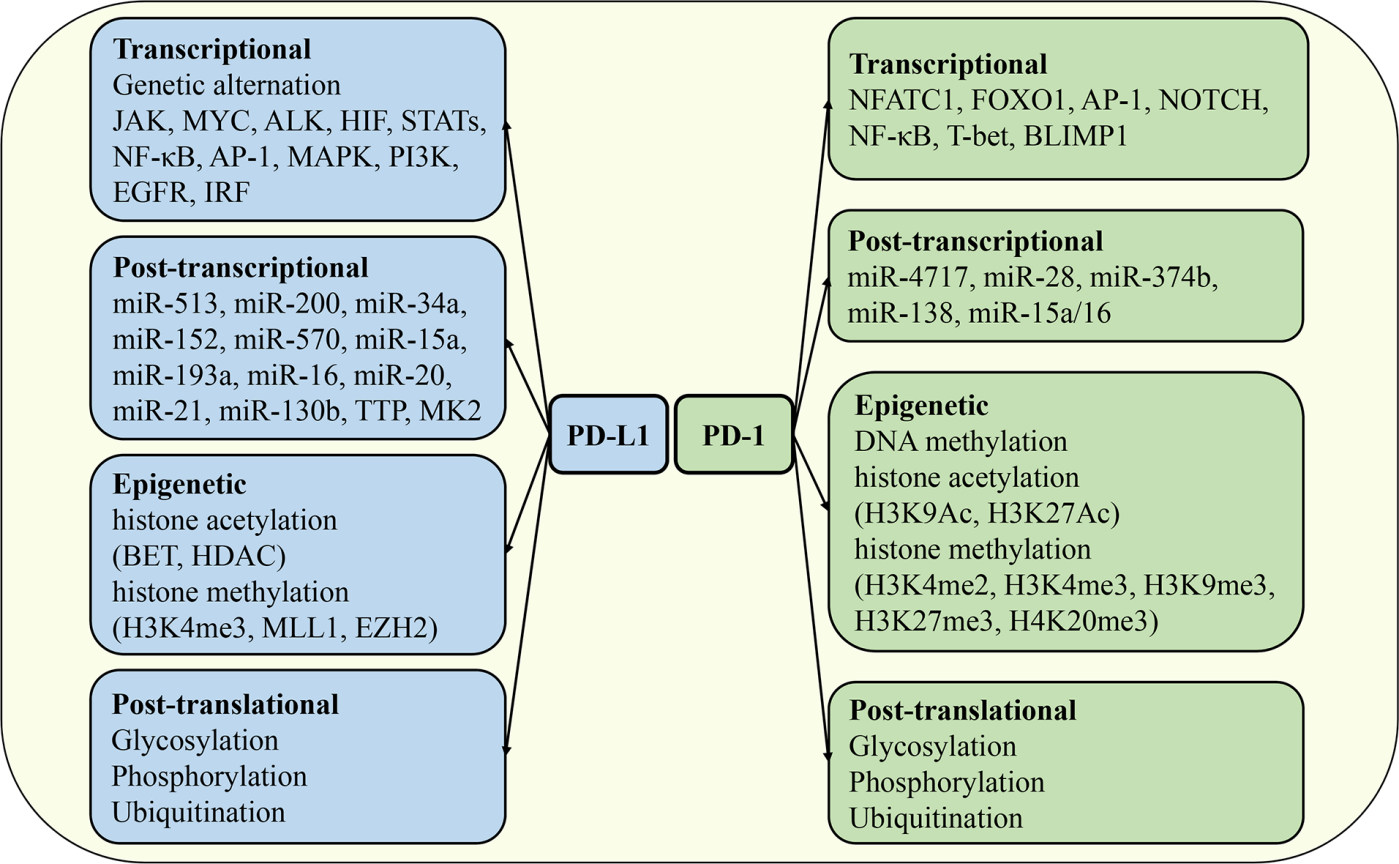
Regulation of PD-1 and PD-L1 expression. The mechanisms regulating the expression of PD-1 and PD-L1 include transcriptional regulation, post-transcriptional regulation, epigenetic regulation, and post-translational modification. JAK, Janus kinase; HIF, hypoxia-inducible factor; STAT, signal transducer and activator of transcription; MAPK, mitogen-activated protein kinases; EGFR, epidermal growth factor receptor; IRF, interferon response factor; FOXO1, forkhead box O1; BLIMP1, B lymphocyte-induced maturation protein 1; TTP, tristetraprolin; BET, bromodomain and extra-terminal domain; HDAC, histone deacetylases; MLL1, mixed lineage leukemia protein 1; EZH2, enhancer of zeste homolog 2.

The expression of PD-1 and PD-L1 is exquisitely regulated in different tissues, cell types, stimulation signals, and immune microenvironments by distinct mechanisms. At the mRNA or protein level, it can be divided into transcriptional regulation, post-transcriptional regulation, epigenetic regulation, and post-translational modification (**Figure 4**). The synergistic operation of the above regulatory mechanisms maintains the dynamic balance of synthesis, modification, degradation, and stability of PD-1 and PD-L1 mRNA and protein, thereby ensuring the operation of PD-1/PD-L1 signaling pathway.

### 5.1 Transcriptional regulation of PD-1 and PD-L1

Genomic alterations, oncogene activation, and inflammatory cytokines can promote PD-L1 expression mediated by a variety of transcription factors. First, genomic alterations can directly or indirectly promote PD-L1 expression, which is closely related to high levels of PD-L1 in tumors. For example, gene rearrangement changes such as amplification and translocation of the *PDCDL1* gene can directly activate the transcriptional program of PD-L1 (87, 88). In addition, when the 3’UTR region of *PDCDL1* is mutated, the expression of PD-L1 protein will be significantly enhanced (89). Moreover, Janus kinase 2 (*JAK2*), which regulates the expression of PD-L1, is located on the same chromosome as *PDCDL1*, and the mutation of JAK family genes can indirectly upregulate the mRNA level of PD-L1 (90). Second, a variety of carcinoma signal and transcription factors such as MYC, ALK, hypoxia-inducing factor 1/2 (HIF-1/2), STAT3, nuclear factor kappa-light-chain-enhancer of activated B cells (NF-κB), activator protein 1 (AP-1), mitogen-activated protein kinases (MAPK), PI3K and epidermal growth factor receptor (EGFR) were involved in the regulation of PD-L1 expression. Among them, the key oncogene MYC is involved in a variety of tumorigenesis processes. Studies have shown that MYC can directly bind to the promoter of the *PDCDL1* gene to activate the transcription of PD-L1 (91). ALK signaling is associated with gene translocation and amplification, and the NPM-ALK fusion protein can activate STAT3 and further bind to the promoter of the *PDCDL1* gene to drive the expression of PD-L1 (92). HIF is a key regulator of tumor angiogenesis and metastasis, and there are hypoxia response elements that can be recognized by HIF in the promoter region of the *PDCDL1* gene (93). NF-κB signaling pathway is involved in the regulation of inflammatory response, and its subunit RELA can also bind to the promoter of *PDCDL1* to promote PD-L1 transcription (94). Finally, various cytokines regulate PD-L1 expression through distinct signal transduction pathways. IFN-γ is the first cytokine found to activate PD-L1 expression and affect anti-tumor immune responses. IFN-γ is mainly derived from activated T cells, which activate the classical JAK/STAT signaling pathway by binding to IFNGR1/2, further inducing the expression of interferon response factor (IRF) and initiating a series of downstream transcriptional programs. It has now been confirmed that IRF1 plays a key role in IFN-γ induced PD-L1 expression (95). In addition, TNF-α, IL-17, IL-1β, IL-10, IL-4, IL-6 and IL-27 derived from various immune cells have been reported to induce the expression of PD-L1 in various immune and non-immune cells under different conditions (49). In addition, TGF-β, as a pleiotropic cytokine, also regulates PD-L1 expression and exhibits cellular and environmental specificity. For example, TGF-β induces PD-L1 expression in DCs cultured *in vitro* but inhibits PD-L1 expression in monocytes (96, 97).

Research on the regulation of PD-1 expression has mainly focused on T cells. At present, several transcription factors have been identified to regulate PD-1 expression on T cells, including the nuclear factor of activated T cells 1 (NFATC1), forkhead box O1 (FOXO1), AP-1, NOTCH, NF-κB, T-bet, and B lymphocyte-induced maturation protein (BLIMP1). During T cell activation, TCR signaling activates the transcription factor NFATC1 through the calcineurin pathway. Nuclear translocation of NFATC1 directly binds to the cis-regulatory elements of the *PDCD1* gene to initiate the PD-1 transcriptional program. Hence, both calcineurin and NFAT inhibitors could inhibit the expression of PD-1 in T cells (98). During chronic viral infection, antigen-specific CD8^+^ T cells gradually transform to an exhausted phenotype and highly express PD-1, which is closely related to the persistent expression of FOXO1. Moreover, specific knockout of FOXO1 in CD8^+^ T cells significantly enhances its antiviral activity (99). In the tumor microenvironment, tumor-infiltrating exhausted T cells also express high levels of PD-1, but AP-1 rather than FOXO1 is the major transcription factor involved in the regulation of PD-1 expression. c-FOS subunit of AP-1 family can target the AP-1 binding site in the promoter of *PDCD1* to promote the transcription of PD-1 (100). In addition to T cells, a few studies have focused on the transcriptional regulation of PD-1 expression in other immune cells. In macrophage activation, Toll-like receptors mediate the activation of the classical NF-κB signaling, and the NF-κB complex further specifically binds to the p65 binding site on the *PDCD1* gene to initiate the transcription of PD-1. In B cells, NFATC1 and NF-κB have also been shown to be involved in the transcriptional regulation of PD-1 (101). In addition to the above-mentioned transcription factors and related signals that promote PD-1 expression, a few transcription factors have been found to inhibit the transcriptional activity of PD-1. BLIMP1 is an important regulator of CD8^+^ T cell differentiation and a key transcription factor that inhibits PD-1 expression. Mechanistically, BLIMP1 not only directly binds to the downstream sequence of the *PDCD1* gene to inhibit the transcription of PD-1, but also indirectly inhibits the expression of PD-1 by downregulating the expression of PD-1 transcriptional activator NFATC1 (102). However, under the condition of chronic infection, although BLIMP1 is highly expressed in exhausted CD8^+^ T cells, its inhibitory effect on PD-1 transcription is abnormal, and the transcription factor T-bet plays a compensatory inhibitory role of PD-1 expression to maintain the function of T cells (103).

### 5.2 Post-transcriptional regulation of PD-1 and PD-L1

MicroRNAs (miRNAs) mediate mRNA degradation or translational repression by specifically recognizing and binding the 3’UTR sequence of mRNA, which is an important mechanism of post-transcriptional regulation. Studies have shown that PD-L1 expression is directly or indirectly regulated by a variety of miRNAs. Among them, miR-513 is the first miRNA identified to specifically regulate PD-L1 expression, which can directly bind to the 3’UTR sequence of *PD-L1* mRNA to inhibit its translation. Interestingly, the expression of miR-513 was inhibited by inflammatory signaling of IFN-γ, a key activator of PD-L1, while overexpression of miR-513 antagonized IFN-γ induced PD-L1 expression (104). In addition, miR-200 blocked the epithelial-mesenchymal transition (EMT) of tumor cells and limited tumor metastasis by targeted inhibition of PD-L1 expression. Zinc finger E-box binding homeobox 1 (ZEB1), a key transcription factor regulating EMT, inhibits the expression of miR-200, so miR-200 is usually at a low level in the tumor microenvironment (105). In addition to miR-513 and miR-200, a variety of miRNAs such as miR-34a, miR-152, miR-570, miR-15a, miR-193a, and miR-16 have been found to directly inhibit the expression of PD-L1 in non-small cell lung cancer, gastric cancer, melanoma, and pancreatic cancer (106). Besides, miR-20, miR-21 and miR-130b could indirectly upregulate the expression of PD-L1 by interfering with protein phosphatase and tensin homolog (PTEN), an inhibitory regulator of PD-L1 (107). Moreover, some kinases are also involved in regulating the mRNA stability and translational program of PD-L1. Tristetraprolin (TTP) interferes with the mRNA stability of PD-L1 by binding to AU-rich elements in the 3’UTR region of the *PDCDL1* gene, while MK2 kinase of MEK signaling pathway can inhibit the expression of TTP indirectly enhance the mRNA stability of PD-L1 and prolong its half-life (108).

PD-1 expression is also regulated by many miRNAs under different conditions. miR-4717 can directly bind to the 3’UTR region of PD-1 mRNA and downregulate its expression to enhance T cell-mediated anti-hepatitis B virus immune response (109). In melanoma, miR-28 inhibits the expression of PD-1 and reverses the T cell exhaustion phenotype, thereby enhancing the release of T cell cytokines such as IL-2 and TNF-α (110). Further studies showed that tumor cells could specifically block the inhibitory effect of miR-28 on PD-1 expression by secreting exosomes carrying CircRNA-002178 (111). In addition, miR-374b and miR-138 could also directly inhibit the translation program of PD-1 mRNA (112, 113). Similar to PD-L1, miRNA can also indirectly regulate PD-1 by controlling the expression of its upstream regulators. In glioma, miR-15a/16 depletion activated tumor-infiltrating CD8^+^ T cells *via* the mammalian target of rapamycin (mTOR) signaling pathway, as indicated by the downregulation of PD-1 expression and the increase of the anti-tumor factor IFN-γ production in T cells (114).

### 5.3 Epigenetic regulation of PD-1 and PD-L1

Epigenetic regulation is also an important mechanism affecting the expression of PD-1 and PD-L1. Histone acetylation and methylation are common epigenetic modifications to regulate PD-L1 expression. Histone acetylation of the *PDCDL1* gene enhances the recruitment and binding of bromodomain and extra-terminal domain (BET) proteins, thereby promoting the transcription of PD-L1. The expression of PD-L1 was significantly downregulated when the interaction between BET protein and *PDCDL1* gene was blocked, while the inhibition of histone deacetylases (HDAC) activity increased the expression of PD-L1 by maintaining the acetylation modification of *PDCDL1* gene (115, 116). In addition, tri-methylation of histone H3 on lysine 4 (H3K4me3) has been reported to be associated with PD-L1 expression. The expression of methyltransferase enhancer of zeste homolog 2 (EZH2) was positively correlated with the level of PD-L1 in lung adenocarcinoma. Moreover, the histone methyltransferase mixed lineage leukemia protein 1 (MLL1) directly binds to the promoter of *PDCDL1* gene and catalyzes H3K4 methylation to upregulate the expression of PD-L1 (117, 118).

PD-1 expression is regulated by DNA methylation and histone modifications. During T cell activation, the PD-1 gene locus shows dynamic DNA methylation characteristics, and the DNA methylation level is negatively correlated with PD-1 expression (119). The *Pdcd1* gene of naive CD8^+^ T cells showed a high level of DNA methylation. In acute lymphocytic choriomeningitis virus (LCMV) infection, the DNA methylation level of the *Pdcd1* gene was decreased with the activation of antigen-specific CD8^+^ T cells, accompanied by the upregulation of PD-1 expression. When the infection resolves, DNA methylation in memory T cells climbs to high levels (119). In experimental autoimmune encephalomyelitis, the PD-1 gene promoter region of autoreactive CD4^+^ T cells was also epigenetically modified (120). The histone modifications that regulate PD-1 expression mainly include methylation and acetylation, which can be divided into promoting or inhibiting modifications. During the activation of CD8^+^ T cells *in vitro*, the expression of PD-1 was up-regulated with the level of active histone modification markers H3K9Ac, H3K27Ac, H3K4me2, and H3K4me3 (102). When the activation of T cells was blocked and the expression of PD-1 was decreased, the repressive histone modification markers H3K9me3, H3K27me3, and H4K20me3 were significantly increased, while the active histone modification markers were decreased (102).

### 5.4 Post-translational regulation of PD-1 and PD-L1

Post-translational modification is the final procedure to regulate the expression level, protein stability, protein translocation, and protein-protein interaction of PD-1 and PD-L1, which directly affects the immune regulatory function mediated by the PD-1/PD-L1 axis. Under different conditions, PD-1 and PD-L1 proteins can undergo post-translational modification reactions such as glycosylation, phosphorylation, and ubiquitination mediated by different factors.

Glycosylation is the most common post-translational modification in mammalian cells. The heavily N-linked glycosylation of the PD-L1 protein significantly increases its stability and prolongs its half-life by about 3-fold by resisting glycogen synthase kinase 3β (GSK3β) mediated 26S proteasome degradation (121). Inhibition of PD-L1 glycosylation in tumor cells can enhance T cell-mediated anti-tumor immune response in breast cancer, suggesting that glycosylation is related to PD-L1 mediated tumorigenic effect (122). Similar to PD-L1, the extracellular IgV domain of PD-1 protein also shows a high degree of N-linked glycosylation, and the structural stability of glycosylated PD-1 protein is significantly increased (123). In addition, a time-dependent increase in glycosylated PD-1 levels was accompanied by a decrease in unglycosylated PD-1 levels during T cell activation (124). Glycosylation modification also affects the interaction between PD-1 and PD-L1. Several *in vitro* and *in vivo* studies have confirmed that interfering with glycosylation modification of PD-1 or PD-L1 will weaken their binding ability, thereby affecting the immunosuppressive effect of PD-1/PD-L1 axis (124, 125).

Phosphorylation is another important post-translational modification. When PD-L1 binds to PD-1, tyrosine phosphorylation of the PD-1 intracellular domain is the basis for initiating downstream inhibitory signals. Phosphorylated PD-1 blocks TCR and CD28 mediated T cell activation signals such as ZAP70, PI3K/AKT, and RAS/MEK/ERK by recruitment of SHP phosphatase (50, 53, 126). At present, the mechanism of PD-1 intracellular phosphorylation and SHP recruitment is not clear, but targeted inhibition of its interaction has been confirmed to enhance T cell-mediated anti-tumor immune response, suggesting the potential possibility of PD-1 phosphorylation as a target for immunotherapy (127). In contrast to phosphorylation in the intracellular domain of PD-1, multiple sites in the extracellular domain of PD-L1 can be phosphorylated. For example, GSK3β induces the phosphorylation of PD-L1 at serine (S184) and threonine (T180) sites and promotes the phosphorylated PD-L1 to the E3 ubiquitin ligase β-transducin repeat-containing protein (β-TrCP), which eventually leads to the degradation of PD-L1 (121). Moreover, phosphorylation of PD-L1 at the serine site (S195) by AMPK kinase induces abnormal PD-L1 glycosylation, which prevents PD-L1 from being transported to the Golgi apparatus for degradation in the endoplasmic reticulum (128).

Ubiquitination is a major post-translational modification that mediates the degradation of PD-1 and PD-L1 proteins. As mentioned above, aberrant PD-L1 glycosylation induces its degradation in the ER, and HRD1 plays an important role in this process as an E3 ubiquitin ligase (128). Interestingly, β-TrCP is involved in the degradation of unglycosylated PD-L1 as a substrate (121). In addition, the ubiquitin ligase STIP1 homology and U-Box-containing protein 1 (STUB1) are also involved in the degradation of membrane-bound PD-L1, while CKLF-like MARVEL transmembrane domain-containing protein 6 (CMTM6) blocked the interaction between STUB1 and PD-L1 to inhibit the degradation of PD-L1 (129). For PD-1, F-box protein 38 (FBXO38) is an important ubiquitin ligase mediating the ubiquitination of PD-1, which can specifically induce the degradation of PD-1 by the proteasome. Knockdown of FBXO38 significantly promoted tumor growth, and its expression was significantly reduced in multiple tumors (130). In addition, two ubiquitin ligases, Casitas B lineage lymphoma (c-Cbl) and kelch-like family member 22 (KLHL22), have also been reported to be involved in regulating the ubiquitination degradation of PD-1. Specifically, c-Cbl initiates the degradation program by binding to the cytoplasmic tail of PD-1, while KLHL22 mediates the ubiquitination degradation of PD-1 before its trafficking to the cell membrane (131, 132).

## 6 Functions of the PD-1/PD-L1 axis

**Figure 5:**
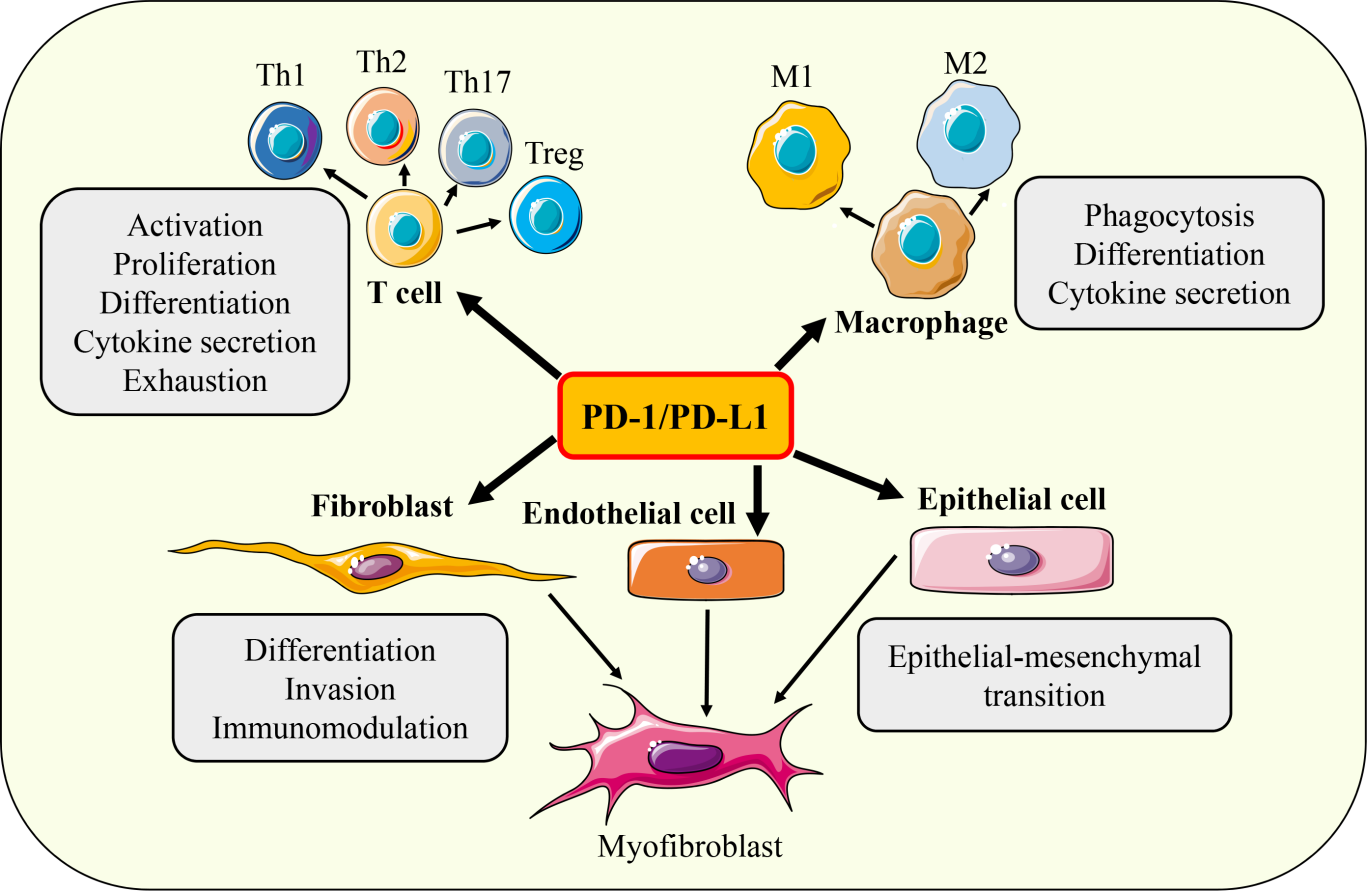
Functions of PD-1/PD-L1 axis. Under different conditions, PD-1/ PD-L1 axis can regulate the activation, proliferation, differentiation, cytokine secretion, exhaustion, phagocytosis, invasion, migration and EMT of T cells, macrophages, fibroblasts, epithelial cells and endothelial cells respectively.

The main function of the PD-1/PD-L1 axis is to limit the excessive activation of T cells to maintain immune tolerance, eliminate inflammatory responses, and prevent tissue damage. It is known that the biological processes of T cell proliferation, differentiation, cytokine secretion, effector function, immune memory, apoptosis, and exhaustion are regulated by PD-1/PD-L1 signaling. In addition, various cellular activities such as activation, phagocytosis, migration, invasion, and EMT of a variety of immune cells (such as B cells, macrophages, DC, NK cells) and non-immune cells (such as fibroblasts, epithelial cells, various tumor cells) have also been found to be regulated by PD-1/PD-L1 axis (**Figure 5**). Next, we mainly introduce the regulation of PD-1/PD-L1 axis on the function of fibrosis-related effector cells such as T cells, macrophages, epithelial cells, and fibroblasts.

### 6.1 Regulation of T cell function by PD-1/PD-L1 axis

The activation of T cells depends on the synergy of two signals, the first signal comes from the binding of the antigen peptide-MHC molecule complex on the surface of APC to the T cell receptor, and the second signal comes from the binding of the co-stimulatory molecules CD80 (B7.1) and CD86 (B7.2) on APC to the T cell stimulatory immune checkpoint CD28. In the absence of the second signal, T cells cannot be fully activated and enter an anergic or tolerant state and are not immunocompetent (133). In the early stage of T cell activation, a large number of negative regulatory factors are upregulated to limit the excessive activation of T cells, which are called immune checkpoint molecules. Cytotoxic T-lymphocyte-associated protein 4 (CTLA-4) is the first identified immune checkpoint that limits T cell activation by competitively binding costimulatory molecules (CD80 and CD86) on the surface of APC to inhibit CD28 signaling (39). PD-1 is another important inhibitory immune checkpoint expressed on the surface of activated T cells. By combining with its ligands PD-L1 and PD-L2, PD-1 antagonizes T cell activation signals such as TCR and CD28 (5). PD-1/PD-L1 pathway plays an important role in regulating the biological processes of T cell activation, differentiation, proliferation, cytokine secretion, and apoptosis, and is a key signal to maintain T cell tolerance and homeostasis (134). Thus, loss of PD-1/PD-L1 signaling causes autoimmune disease, whereas hyperactivated PD-1 signaling leads to decreased immune surveillance. In the tumor microenvironment, tumor cells upregulate the expression of PD-L1 and engaged with PD-1 on the surface of T cells, further inducing the exhaustion of CD8^+^ T cells and weakening their tumor-killing ability, so as to achieve immune escape (135). Subsequent clinical trials have confirmed that targeted inhibition of the PD-1/PD-L1 signaling pathway can continuously activate anti-tumor immune response, inhibit tumor growth, and reduce the disease burden of patients. Therefore, in 2014, the US Food and Drug Administration approved pembrolizumab, the first monoclonal antibody targeting PD-1, for immunotherapy in patients with unresectable or metastatic melanoma (136). The application of immune checkpoint inhibitors has significantly improved the clinical therapeutic ability of many tumors, and further confirmed the key role of PD-1/PD-L1 signal in tumorigenesis. Subsequently, a variety of immune checkpoint inhibitors targeting PD-1/PD-L1 axis, such as nivolumab, atezolizumab, avelumab, and durvalumab, were approved for the treatment of non-small cell lung cancer, melanoma, renal cell carcinoma, bladder cancer, Hodgkin’s lymphoma, liver cancer, and gastric cancer (137). Although some patients are resistant to PD-1/PD-L1 inhibitors, the overall clinical efficacy is still remarkable. Therefore, the PD-1/PD-L1 axis is a key regulator of T-cell immunity. Targeted inhibition or activation of the PD-1/PD-L1 signaling pathway can improve the dysfunction of T cell-dependent adaptive immunity, restore immune homeostasis, and thus treat immune-related diseases.

### 6.2 Regulation of macrophage function by PD-1/PD-L1 axis

Macrophages are involved in complex physiological and pathological immune responses with their highly plastic and versatile immunomodulatory abilities. In the tumor microenvironment, tumor-associated macrophages (TAMs) can inhibit the anti-tumor effector function of T cells and enhance the immune escape ability of tumor cells by hijacking the PD-1/PD-L1 signaling pathway. On the other hand, the phenotype and function of TAMs are also precisely regulated by PD-1/PD-L1 signaling, underscoring the importance of the PD-1/PD-L1 axis for macrophage immunity. PD-1/PD-L1 signaling regulates macrophage immune responses through multiple pathways such as controlling macrophage phagocytosis, cytokine production, and polarization. Phagocytosis is a key immune effector function of macrophages. The expression of PD-1 in TAMs was significantly upregulated, and the volume of PD-1^+^ TAMs increased and appeared as foam cells. Further studies found that the phagocytosis ability of PD-1^+^ TAMs was significantly reduced while blocking PD-1/PD-L1 signaling could promote the phagocytosis of macrophages to tumor cells and enhance the macrophage dependent anti-tumor immune response (138, 139). Secretion of cytokines is one of the mechanisms by which macrophages exert immunomodulatory effects. In RAW264.7 macrophages, LPS-induced IL-12 production could be inhibited by PD-1 signaling, which was similar to the inhibition of PD-1 mediated T cell activation by recruiting SHP-2 phosphatase to inhibit the activation of JNK/PI3K/AKT pathway (140). In chronic hepatitis C virus infection, PD-1/PD-L1 signaling can also downregulate the expression of IL-12 by interfering with STAT1 pathway (141). The function of macrophages changes with their phenotypic switching. Among them, M1 macrophages mainly play a pro-inflammatory role, while M2 macrophages mainly play an immunosuppressive function. Studies have shown that TAMs usually upregulate M2 macrophage related markers such as CD206 and CD11c, accompanied by high expression of PD-1, suggesting that PD-1/PD-L1 signaling may be involved in M2 macrophage polarization (138). When PD-1 was specifically knocked out, macrophages were activated to the M1 type and secreted a variety of pro-inflammatory factors (139). In addition, PD-1/PD-L1 signaling can also interfere with the ability of activated macrophages to mediate adaptive immune cells by inhibiting the expression of costimulatory molecules CD80 and CD86 and MHC molecules on the surface of activated macrophages (140). In conclusion, PD-1/PD-L1 signaling regulates macrophage function through multiple pathways to maintain immune homeostasis.

### 6.3 Regulation of epithelial cell function by PD-1/PD-L1 axis

The transition of epithelial cells to mesenchymal cells (EMT), which in turn replenish the number of myofibroblasts, is a key cellular event in the fibrotic response. Meanwhile, the occurrence of EMT is also closely related to tumor metastasis. At present, the correlation between PD-L1 and EMT has been confirmed in a variety of tumors such as non-small cell lung cancer, breast cancer, and head and neck squamous cell carcinoma, and the existing evidence suggests that there may be bidirectional feedback regulation between PD-L1 and EMT (142). Clinical data show that PD-L1 is highly expressed in stromal cells and lowly expressed in epithelial cells in a variety of tumor tissues. Moreover, the level of PD-L1 was positively correlated with the expression of EMT markers such as Snail, ZEB1, Vimentin, and N-cadherin, and negatively correlated with the expression of epithelial marker E-cadherin (143, 144). The above evidence provides sufficient evidence for a link between PD-L1 and EMT, but cannot confirm the temporal relevance of their regulatory relationship. At this point, researchers have carried out a large number of studies to reveal this puzzle. The adaptor protein Crk plays an important role in promoting the expression of EMT markers ZEB1 and N-cadherin. When Crk was knocked out specifically, the expression of PD-L1 was significantly inhibited (145). In addition, MUC1 can induce EMT by activating the NF-κB/ZEB1 pathway, and the p65 subunit of NF-κB can directly bind to the promoter of PD-L1 to promote its transcription (146). TGF-β, as a key inducer of EMT, can also upregulate the expression of PD-L1 by activating PI3K/AKT and MEK/ERK signaling pathways (147). Together, these studies suggest that key signals regulating EMT can simultaneously affect PD-L1 expression. On the other hand, the upregulation of PD-L1 can also induce EMT. In glioblastoma, PD-L1 upregulates the expression of EMT markers N-cadherin and vimentin by activating transcription factors Slug and β-catenin (148). In addition, the expression of PD-L1 was correlated with the migration of renal cancer cells, and silencing of PD-L1 significantly inhibited the expression of EMT markers vimentin and F-actin. Overexpression of PD-L1 promotes EMT by activating transcription factor sterol-regulatory element binding protein 1 (SREBP1) (149). In conclusion, the expression of PD-L1 is closely related to the occurrence of EMT, and multiple signals that initiate the EMT program can promote the expression of PD-L1, while upregulated PD-L1 can also drive the EMT process.

### 6.4 Regulation of fibroblast function by PD-1/PD-L1 axis

In fibrotic response, fibroblasts are the main source of myofibroblasts. The activation, invasion, and immunoregulatory function of fibroblasts are also closely regulated by the PD-1/PD-L1 signaling pathway. TGF-β is a key effector to induce the transdifferentiation of fibroblasts into myofibroblasts. Many studies have confirmed that TGF-β can induce the upregulation of PD-L1 during the activation of fibroblasts, and PD-L1 mediated signaling is necessary for fibroblast activation. In primary human lung fibroblasts, TGF-β induces PD-L1 expression and promotes transdifferentiation depending on SMAD3, p38, and yes-associated protein/transcriptional co-activator with PDZ-binding motif (YAP/TAZ) signaling. After silencing PD-L1 by siRNA, the expression levels of α-SMA, collagen I, and fibronectin in fibroblasts were significantly decreased (150). Mechanically, PD-L1 acts as a cofactor and binds to the key transdifferentiation transcription factor SMAD3 to form a protein complex, and then directly activates the transcription of transdifferentiation genes such as α-SMA. On the other hand, PD-L1 is involved in mediating the transduction of GSK3β/β-catenin signaling to indirectly promote the activation of fibroblasts (150). Another study found that PD-L1 promoted TGF-β induced fibroblast transdifferentiation by impairing autophagy through activation of PI3K/AKT/mTOR signaling pathway, and inhibition of PD-L1 using monoclonal antibody prevented fibroblast activation by restoring autophagy activity (151). Therefore, PD-L1 may act as an intermediate to coordinate the activities of multiple signaling pathways related to transdifferentiation to regulate the activation of fibroblasts. In addition, PD-L1 expression is also a key factor in maintaining the invasive phenotype of fibroblasts. Invasive fibroblasts with high expression of PD-L1 in lung tissue of patients with idiopathic pulmonary fibrosis (IPF) are closely related to the progression of pulmonary fibrosis. Overexpression of PD-L1 in normal fibroblasts enhances their invasion and migration abilities, and injection of invasive fibroblasts into immunodeficient mice induces severe pulmonary fibrosis (152). In addition, fibroblasts can regulate T-cell immune responses by expressing PD-L1. *In vitro* studies have shown that TGF-β can not only upregulate the expression of PD-L1 on the cell membrane during the activation of fibroblasts, but also induce fibroblasts to secrete exosomes containing PD-L1, and PD-L1^+^ exosomes can inhibit the proliferation of T cells (153). *In vivo* studies have also found that the upregulation of PD-L1 on fibroblasts in the fibrotic lung can induce the exhaustion of T cells and promote the formation of immunosuppressive microenvironment (154). In conclusion, PD-1/PD-L1 signaling can directly or indirectly participate in the fibrotic response by regulating the activation of fibroblasts and their immunomodulatory capacity.

### 6.5 Regulation of endothelial cell function by PD-1/PD-L1 axis

Similar to epithelial cells, endothelial cells can directly transform into myofibroblasts under specific stimuli or, by modulating immune responses, participate in fibrosis. However, only a few studies have focused on the role of endothelial cell-mediated PD-1/PD-L1 signaling in fibrosis. In radiation therapy induced pulmonary fibrosis, PD-L1^+^ endothelial cells co-express TGF-β. Bintrafusp alfa, a bifunctional inhibitor of PD-L1 and TGF-β, significantly ameliorated radiation therapy induced pulmonary fibrosis (155). These results tentatively suggest that endothelial cell mediated PD-L1 signaling is profibrotic, they can induce an immunosuppressive microenvironment while directly activating a profibrotic response by upregulating the expression of PD-L1 and TGF-β. However, in pulmonary arterial hypertension (PH) associated fibrosis, the maintenance of PD-L1 expression in right ventricular vascular endothelial cells by Treg is important for the inhibition of fibrosis. Once Treg was depleted, the expression of PD-L1 in endothelial cells was downregulated, and the inflammation and fibrosis of the right ventricle were significantly aggravated. Moreover, blockade of PD-1/PD-L1 signaling could offset the protective effect of Treg on PH-induced fibrosis (156). These evidences suggest that Treg can suppress inflammation and fibrosis by inducing PD-L1 expression in endothelial cells. Interestingly, murine vascular endothelial cells can induce allogeneic Treg generation in a PD-L1-dependent manner, and these induced Tregs can inhibit the proliferation of alloreactive T cells (157). In many cases, Tregs promote fibrosis progression by releasing immunosuppressive and profibrotic factors such as IL-10 and TGF-β, which suggests that endothelial cells can indirectly promote fibrosis by inducing Treg activation through PD-L1 signaling. In a murine heart allograft model, blockade of Lymphotoxin-Beta Receptor downregulated PD-L1 expression in vascular endothelial cells and induced cardiac inflammation and fibrosis (158). This further indicates that the expression of PD-L1 in endothelial cells is important for the maintenance of immune tolerance. In the early stage of lung injury induced by SARS-CoV-2, pulmonary venules and capillaries have obvious proliferation, dilatation, and distortion, accompanied by a significant upregulation of PD-L1 expression in endothelial cells. With the aggravation of infection, lung tissue injury gradually develops to fibrosis, while endothelial cells still express high levels of PD-L1 (159). This also suggests that PD-L1 signaling in endothelial cells is associated with fibrosis development, but the molecular mechanisms still need to be further elucidated. Taken together, endothelial cell-mediated PD-1/PD-L1 signaling is also an important regulator of fibrogenesis, and its specific role may be organ or microenvironment determined.

## 7 PD-1/PD-L1 axis in organ fibrosis

Fibrosis is a pathological response finely regulated by the immune system, and its occurrence and development involve the participation of various immune cells and various immune regulatory factors. Studies have shown that PD-1/PD-L1 signaling mediates fibrotic pathological responses by regulating T cell immunity, fibroblast activation, and epithelial EMT. However, the clinical and experimental data on PD-1/PD-L1 axis in fibrotic diseases are still limited.

### 7.1 PD-1/PD-L1 axis in pulmonary fibrosis

At present, the research on PD-1/PD-L1 axis in pulmonary fibrosis mainly focuses on IPF and bleomycin-induced experimental pulmonary fibrosis. First, PD-1/PD-L1 signaling modulates pulmonary fibrosis by regulating T cell function. The expression of PD-1 in lung tissue and peripheral blood T cells of patients with IPF is significantly increased, while the level of PD-L1 has no significant change compared with healthy controls (160). The proliferation of PD-1^+^CD4^+^ T cells was significantly reduced, but their ability to secrete pro-fibrotic factors such as IL-17A and TGF-β was significantly increased, which could induce collagen I production from lung fibroblasts *in vitro*. Notably, pro-fibrotic T cells were mainly Th17 subtypes, which were induced by the PD-1/STAT3 pathway. In addition, PD-1 knockout or anti-PD-L1 antibodies significantly inhibited bleomycin-induced pulmonary fibrosis (161). The above evidence suggests that PD-1 signaling induces the activation of T cells with a profibrotic phenotype that plays an important role in the pathogenesis of IPF.

Second, PD-L1 expression correlates with the immunomodulatory capacity of lung fibroblasts. As a member of the AP-1 family, JUN is an important transcription factor regulating cell growth and proliferation, and excessive activation of JUN can induce spontaneous pulmonary fibrosis. In JUN-induced pulmonary fibrosis, the high expression of JUN in fibroblasts induces the upregulation of PD-L1 and CD47 by regulating chromatin remodeling and enhancing DNA accessibility and promoting the release of pro-fibrotic factor IL-6 (154). Activated fibroblasts with high expression of PD-L1 and CD47 can further interact with T cells and macrophages in lung tissue, induce the exhaustion of T cells and inhibit the clearance of myofibroblasts by macrophages, thereby promoting the formation of an inhibitory immune microenvironment (154). Combined blockade of PD-L1, CD47, and IL-6 signaling can significantly inhibit bleomycin-induced pulmonary fibrosis in mice by activating adaptive immune response and macrophage phagocytic activity, suggesting that PD-L1 mediated pro-fibrotic response may be the common mechanism of pulmonary fibrosis induced by different factors (154). Another *in vitro* study found that TGF-β induced PD-L1 expression on the cell surface and exosomes of multiple human and mouse derived fibroblasts by activating the classical SMAD2/3 and the non-classical YAP/TAZ pathways, and TGF-β-activated fibroblasts derived exosomes can inhibit the proliferation of T cells (153). These results suggest that PD-L1 can mediate the immunomodulatory function of fibroblasts to promote fibrosis.

Third, PD-L1 directly regulates the activation of fibroblasts and their profibrotic phenotype. Invasive fibroblasts derived from the lung tissues of IPF patients highly express PD-L1, which is closely related to the progression of pulmonary fibrosis (152). Overexpression of PD-L1 in normal fibroblasts significantly enhanced their invasion and migration abilities, indicating that PD-L1 expression is a key factor in acquiring the invasive phenotype of fibroblasts. Mechanistically, the expression of PD-L1 and the invasive ability of invasive fibroblasts is regulated by p53 and focal adhesion kinase (FAK) signals. Injection of invasive fibroblasts into immunodeficient mice *via* the tail vein can induce severe pulmonary fibrosis, and blockade of PD-1/PD-L1 signaling can inhibit pulmonary fibrosis (152). Relevant *in vitro* studies have shown that silencing PD-L1 can inhibit TGF-β induced transdifferentiation of pulmonary fibroblasts and reduce their migration ability (150). In particular, the expression of PD-L1 in fibroblasts is dependent on TGF-β mediated activation of SMAD3 and p38 signaling. Upregulated PD-L1 acts as a SMAD3 cofactor to initiate transcription of transdifferentiation related gene α-SMA, and activates GSK3β/β-catenin signaling pathway to promote transdifferentiation (150). In addition, anti-PD-L1 antibodies can restore the impaired autophagy activity of fibroblasts under the stimulation of TGF-β and inhibit their transdifferentiation by inhibiting the PI3K/AKT/mTOR pathway (151). Thus, PD-L1 can regulate the fibrotic response by directly controlling the activation of key fibrotic effector cells, fibroblasts.

Fourth, sPD-L1 may be an important profibrotic factor. In clinical studies of IPF, researchers found that plasma levels of sPD-L1 were significantly higher in IPF patients than in healthy controls, and that sPD-L1 levels tended to increase with the progression of fibrosis (162, 163). As mentioned above, certain forms of sPD-L1 are fully immunocompetent and can directly activate PD-1 signaling to exert immunosuppressive function. Thus, sPD-L1 originating from fibrotic lung tissue may be transmitted distally through the circulation, thereby creating a systemic immunosuppressive environment for fibrosis progression. However, further preclinical *in vivo* studies are needed to confirm this hypothesis. In addition, which effector cells are responsible for the large amount of secreted sPD-L1 also needs to be elucidated.

Fifth, interactions between the lung microbiome and PD-1/PD-L1 signaling may also influence the progression of pulmonary fibrosis. Although it has been considered that the lung is sterile, a large number of evidences have shown that local lung microbiome integrity is important for maintaining lung homeostasis (164). In addition, the abnormal diversity and composition of the lung microbiome are associated with a variety of lung diseases (165). During the formation stage of lung microbiome in neonatal mice, changes in respiratory microbial subsets can lead to up-regulation of PD-L1 expression in lung CD11b^+^ DC and induce the generation of Helios^−^ Treg, thereby protecting neonatal mice from airway hyperresponsiveness to allergens (166). In lung cancer, Lee et al. found that the microbial species in bronchoalveolar lavage fluid of cancer patients were correlated with the PD-L1 level. *Veillonella dispar* was identified in the group with high PD-L1 expression, and *Neisseria* was mainly in the group with low PD-L1 expression (167). Furthermore, Kumanogoh et al. found that the presence of the lung microbiome is critical for supporting patient sensitivity to anti-PD-1 therapy (168). The above evidence suggests that there is a reciprocal regulatory relationship between PD-1/PD-L1 axis and the lung microbiome, and their interaction is of great significance for the homeostasis of the lung immune microenvironment, but its effect on pulmonary fibrosis still needs to be further explored.

Based on the above evidence, PD-1/PD-L1 signaling appears to promote the progression of pulmonary fibrosis. Similar to our view, Jiang et al. in their recent review of the role of PD-1/PD-L1 axis in IPF concluded that the PD-1/PD-L1 pathway mainly plays a profibrotic role (169). However, PD-1/PD-L1 signaling may also prevent fibrosis development by correcting the dysregulated pulmonary immunity. Resting mesenchymal stem cells (MSCs) express a low level of PD-L1, and when they interact with activated T cells, they can significantly upregulate the expression of PD-L1 to inhibit inflammatory response, suggesting that MSCs can rely on PD-1/PD-L1 axis to exert their immunomodulatory function (160). In a humanized mouse model, intravenous administration of human MSCs inhibited pathological T cell infiltration and proinflammatory cytokine production in mouse lung tissue and attenuated bleomycin-induced pulmonary fibrosis, while PD-L1 monoclonal antibody significantly interfered with these therapeutic effects (160). In other words, MSCs inhibited the inflammatory response in lung tissue by activating the PD-1/PD-L1 signaling pathway, thereby reducing fibrosis. This also suggests the theoretical feasibility of activating PD-1/PD-L1 signaling to inhibit inflammatory response for the treatment of pulmonary fibrosis.

In silicotic fibrosis induced by silica exposure, a few studies have begun to focus on the role of PD-1/PD-L1 pathway. Two clinical studies of silicosis patients conducted in China and Brazil obtained similar results. The expression of PD-1 on CD4^+^ and CD8^+^ T cells and the expression of PD-L1 and PD-L2 on CD14^+^ monocytes in peripheral blood of silicosis patients are significantly lower than those of healthy people, and the downregulation of PD-1 may be related to the single nucleotide polymorphism of *PDCD1* gene (170, 171). However, our previous experimental study on silicosis found that the overall levels of PD-1 and PD-L1 were significantly increased in the lung tissues, spleen, and lymph nodes of silicosis mice (172). In-depth analysis of T cell subtypes found that the expression of PD-1 in CD8^+^ T cells was upregulated in the peripheral blood of early silicosis mice, but downregulated in the lung tissue. The expression of PD-1 in CD4^+^ T cells was significantly increased in lung tissues of early and late silicosis, but there was no significant change in peripheral blood (172). Based on this, we further blocked the PD-1/PD-L1 pathway in an experimental silicosis model, and the results showed that small molecule inhibitors of PD-1/PD-L1 could significantly reduce silicosis fibrosis (172). Hence, PD-1/PD-L1 signaling is also involved in the pathogenesis of silicosis, but the mechanism needs to be further studied *in vivo* and *in vitro*.

In conclusion, PD-1/PD-L1 axis plays a key regulatory role in the pathogenesis of pulmonary fibrosis induced by different factors, and its effects is dominated by pathogenic factors, fibrosis stage and immune cell types (**Table 1**). The wide expression of PD-1 and PD-L1 identifies its versatile immunomodulatory ability, which can participate in pulmonary fibrosis by regulating a variety of biological activities such as T cell differentiation and cytokine secretion, fibroblast activation, and the interaction between various fibrotic effector cells. Overall, PD-1/PD-L1 axis is mainly pro-fibrotic, but it can also inhibit fibrosis progression through specific mechanisms. Targeting the PD-1/PD-L1 pathway may be a new direction for immunotherapy of pulmonary fibrosis in the future. However, the unique role of PD-1/PD-L1 axis in different scenarios of pulmonary fibrosis should be further explored.

**Table 1:** Studies of PD-1/PD-L1 signaling in pulmonary fibrosis.

	Disease or Model	Cell type	Level	Species	Main findings	Ref
**PD-L1**	IPF & JUN induction	Fibroblasts	Up	Human & Mouse	PD-L1 is upregulated in fibrotic lung tissue, expression of PD-L1 in fibroblasts can interact with T cells and macrophages to induce immunosuppression and profibrotic lung microenvironment.	(154)
	IPF & Fibroblasts induction	Fibroblasts	Up	Human & Mouse	PD-L1 was upregulated on invasive lung fibroblasts and was required for the invasive phenotype of lung fibroblasts, activating PD-L1 in IPF fibroblasts promoted invasion *in vitro* and pulmonary fibrosis *in vivo*.	(152)
	IPF & Bleomycin induction & TGF-β induction	Fibroblasts	Up	Human & Mouse	Upregulation of PD−L1 in the lungs of IPF patients and mice with pulmonary fibrosis induced by bleomycin and TGF−β were detected. Fibroblast PD−L1 may promote pulmonary fibrosis through both Smad3 and β−catenin signaling.	(150)
	Bleomycin induction & TGF-β induction	Fibroblasts	Up	Mouse	PD-L1 is highly expressed in the fibrotic lung tissue. The anti-PD-L1 antibody significantly alleviated bleomycin-induced lung fibrosis in mice and inhibited the proliferation, migration, activation of TGF-β1 induced lung fibroblasts, through reverse the impairment of autophagy.	(151)
	IPF & TGF-β induction	Fibroblasts	Up	Human & Mouse	PD-L1 levels were higher in IPF fibroblasts than in healthy controls. TGFβ induces the expression of PD-L1 in human and murine fibroblasts in a Smad2/3- and YAP/TAZ dependent manner. TGFβ induced fibroblast extracellular vesicles contain PD-L1 which can inhibit T cell proliferation and activated fibroblast migration.	(153)
	PF & Bleomycin induction	MSCs		Human & Mouse	PD-L1 expressing human MSCs could alleviate pulmonary fibrosis and improve lung function by suppressing bleomycin-induced human T-cell infiltration and proinflammatory cytokine production through PD-1/PD-L1 pathway.	(155)
	Silicosis & Asbestosis	CD14^+^ monocytes	Down	Human	The expression of PD-L1 on CD14^+^ monocytes in peripheral blood of patients with silicosis and asbestosis was significantly lower than that of healthy controls.	(161)
	Silicosis	CD4^+^/CD8^+^ T cells	Up/ Down	Mouse	PD-L1 expression was upregulated in CD4^+^ T cells in lung tissue of silicosis mice but did not reach statistical significance, while it was downregulated in CD8^+^ T cells.	(162)
**sPD-L1**	IPF		Up	Human	Plasma levels of sPD-L1 were significantly higher in IPF patients than in healthy controls, and that sPD-L1 levels tended to increase with the progression of fibrosis	(157) (158)
**PD-1**	IPF & JUN induction	T cells & Macrophages	Up	Human & Mouse	The level of PD-1 is positively correlated with the immunosuppressive phenotype of T cells and macrophages in the lungs.	(154)
	IPF & Sarcoidosis & Bleomycin induction	CD4^+^ T cells	Up	Human & Mouse	PD-1^+^CD4^+^ T cells with reduced proliferative capacity and increased TGF-β/IL-17A expression were detected in IPF, sarcoidosis, and bleomycin CD4^+^ T cells. PD-1^+^ Th17 cells are the predominant CD4^+^ T cell subset expressing TGF-β. Coculture of PD-1+CD4^+^ T cells with human lung fibroblasts induced collagen-1 production.	(156)
	PF	T cells & Lung tissue	Up	Human	Upregulation of PD-1 expression was found in circulating T cells and lung tissues of patients with pulmonary fibrosis.	(155)
	Silicosis	CD4^+^/CD8^+^ T cells	Down	Human	PD-1 expression is significantly reduced in both peripheral CD4^+^ and CD8^+^ T cells of silica-exposed workers.	(157)
	Silicosis & Asbestosis	CD4^+^/CD8^+^ T cells	Down	Human	PD-1 was expressed at significantly lower levels on CD4^+^ and CD8^+^ peripheral T cells from patients with asbestosis and silicosis than on cells from healthy controls. Decreased PD-1 expression on CD4^+^ or CD8^+^ T cells in peripheral blood was positively correlated with the asbestosis severity.	(161)
	Silicosis	CD4^+^/CD8^+^ T cells	Up/Down	Mouse	PD-1 expression was significantly upregulated in CD4^+^ T cells in lung tissues of mice with early and late silicosis, while it was downregulated in CD8^+^T cells in lung tissues of mice with early silicosis.	(160)

PD-L1, programed death ligand 1; sPD-L1, soluble PD-L1; PD-1, programmed cell death protein 1; IPF, idiopathic pulmonary fibrosis; PF, pulmonary fibrosis; TGF-β, transformation growth factor; MSCs, mesenchymal stem cells; YAP/TAZ, yes-associated protein/transcriptional co-activator with PDZ-binding motif; IL-17A, interleukin 17A.

### 7.2 PD-1/PD-L1 axis in liver fibrosis

PD-1/PD-L1 axis is also involved in liver fibrosis, liver cancer-related fibrosis, and cirrhosis. In hepatitis B virus (HCV) infection-induced liver fibrosis, the expression of PD-1 and PD-L1 in liver tissue is significantly increased, which together with other immune checkpoint molecules such as CTLA-4, T cell immunoglobulin and mucin domain-containing protein 3 (TIM-3) and lymphocyte activation gene 3 (LAG3), promotes the formation of immunosuppressive microenvironment in liver (173). In addition, there is a positive correlation between the levels of PD-1 and PD-L1 and the severity of liver fibrosis, suggesting that PD-1/PD-L1 signaling may promote liver fibrosis. Another clinical study also found that serum sPD-1 levels in patients with HCV infection gradually increased with the severity of liver fibrosis, and was significantly higher than that in healthy people (174). Experimental studies have shown that Golgi membrane protein 1 (GOLM1) is significantly upregulated in carbon tetrachloride (CCL_4_) induced mouse liver fibrosis, and GOML1 induces PD-L1 expression and promotes fibrosis by activating EGFR/AKT/STAT3 signaling pathway (175). The aggressive fibrotic response further induces a suppressive immune microenvironment, thereby accelerating hepatocellular carcinoma (HCC) development. Therefore, targeted inhibition of PD-L1 can enhance anti-tumor and anti-fibrotic immune responses by reducing M2 macrophages and myeloid-derived suppressor cells in the fibrotic immunosuppressive microenvironment, and increasing the number of effector CD8^+^ T cells in liver fibrosis induced HCC (175). In liver cirrhosis, the expression of PD-1 upregulated in liver, peripheral blood, and spleen T cells, and the blockage of liver macrophages phagocytosis mediated by PD-1/PD-L1 pathway may be involved in the pathogenesis of liver cirrhosis (176). The above research evidence collectively proves that PD-1/PD-L1 signaling promotes the development of liver fibrosis, but other studies have found that indirect activation of PD-L1 signaling can prevent liver fibrosis. In CCL_4_-induced liver fibrosis in mice, kinsenoside can inhibit the activation and maturation of DCs by targeting the PI3K/AKT/FoxO1 signaling pathway and upregulating the expression of PD-L1 in DCs while inhibiting the release of IL-12 (177). Expression of PD-L1 on the surface of DCs interacts with CD8^+^ T cells to inhibit their activation, while the reduction of IL-12 leads to the blockade of hepatic stellate cell activation and ECM release, thereby reducing liver fibrosis (177). In summary, similar to pulmonary fibrosis, PD-1/PD-L1 axis also plays an important role in the pathogenesis of liver fibrosis. Notably, PD-1/PD-L1 axis mediated immunomodulation can regulate the development of liver fibrosis by reprogramming the function of liver macrophages, DC, and T cells. Similar to pulmonary fibrosis, the massive proliferation of myofibroblasts is the direct cause of the aggravation of liver fibrosis. However, the role of PD-1/PD-L1 signaling in the transdifferentiation of hepatic stellate cells into myofibroblasts has been poorly studied, so this is the focus of further attention, and the mechanism needs to be further elucidated.

### 7.3 PD-1/PD-L1 axis in tubulointerstitial nephritis and renal fibrosis

Tubulointerstitial nephritis (TIN) is one of the major causes of acute kidney injury and may eventually lead to chronic kidney disease such as renal fibrosis. Although not very common, there have been some case reports of TIN and renal fibrosis development during the treatment of PD-1 or PD-L1 inhibitors against a variety of cancers (178). In a clinical application of Nivolumab in the treatment of recurrent gastric cancer, the patient developed an apparent acute granulomatous TIN. Renal biopsy showed that the main lesions occurred in the renal tubular and interstitial areas, and renal tubular atrophy with granulomatous changes was the typical feature. Notably, PD-L1 was highly expressed by both macrophages in the granulomatous tissue and degenerated renal tubular epithelial cells (179). Moreover, TIN also occurs in patients with metastatic malignant melanoma treated with CTLA-4 and PD-1 inhibitors. Histological examination revealed inflammatory cell infiltration, edema and fibrosis in the renal interstitium. Among the infiltrated cells, CD3^+^ T cells and CD163^+^ macrophages were predominant, which highly expressed PD-L1 and were negative for PD-1 (180). This suggests that T cells and macrophages play an important role in renal fibrosis caused by PD-1/PD-L1 signaling imbalance. In addition, in the treatment of metastatic renal cell carcinoma with atezolizumab, both CD4^+^ and CD8^+^ T cells are key players in TIN pathogenesis (181). The above clinical data have fully proved that PD-1/PD-L1 axis is related to TIN and renal fibrosis, while other experimental evidence may partly explain its mechanism. Renal tubular epithelial cells (TEC) are a special type of APC in the kidney, which constitutively express MHC-II molecules and weakly express PD-L1 under physiological conditions (182, 183). When stimulated by pro-inflammatory factors such as IFN-γ, the expression of PD-L1 in TEC was increased in a dose-dependent manner. Moreover, blocking PD-1/PD-L1 signaling in IFN-γ-activated TEC and T cell co-culture system could relieve the inhibitory effect of TEC on Th1 and Th2 cytokine production (182). Interestingly, TGF-β, a crucial profibrotic factor, downregulated PD-L1 expression in TEC and enhanced their ability to activate CD8^+^ T cells (184). This suggests that TGF-β may promote TIN generation by regulating the interaction between TEC and T cells, and then induces renal fibrosis. Mechanistically, PD-1/PD-L1 axis may mediate TIN and renal fibrosis from the following aspects. First, under the use of immune checkpoint inhibitors, the immune system, especially T cells, is overactivated, which leads to the initiation of inflammatory response, resulting in adverse effects. Thus, TIN and even renal fibrosis induced by PD-1 or PD-L1 inhibitors may represent an autoimmune response. Second, the physiological expression of PD-L1 in renal tubular epithelial cells is important for the maintenance of immune tolerance in renal interstitium. The application of PD-1 or PD-L1 inhibitors disrupts renal interstitial immune homeostasis, which leads to T cell overactivation and ultimately TIN. In the interaction between renal tubular epithelial cells and T lymphocytes, TEC can present antigens to initiate T cell activation, but on the other hand, it can inhibit T cell activation by binding PD-L1 to the PD-1 receptor on the surface of T cells. Third, it is worth noting that in TIN induced by PD-1 or PD-L1 inhibitors, patients are usually exposed to some drugs with nephrotoxicity such as proton pump inhibitors and non-steroidal anti-inflammatory drugs before receiving immune checkpoint inhibitor therapy. This suggests that PD-1 or PD-L1 inhibitors may have reactivated drug-specific effector T-cell clones in the resting state. In conclusion, the PD-1/PD-L1 axis is important for the pathogenesis of renal fibrosis.

### 7.4 PD-1/PD-L1 axis in other fibrosis

PD-1/PD-L1 axis is also involved in other fibrotic diseases with relatively low incidence. In systemic sclerosis associated fibrosis, the PD-1 level of Treg and γδT cells in peripheral blood of patients were significantly increased, and PD-1 co-expressed with another inhibitory immune checkpoint T cell immunoreceptor with Ig and ITIM domains (TIGIT) (185). In addition, compared with TIGIT and TIM-3, PD-1 plays a major role in the secretion of peripheral T cell cytokines in patients with systemic sclerosis (185). In upper airway tracheal stenosis associated fibrosis, PD-1 and PD-L1 expression were significantly upregulated in cricotracheal resection tissues of patients with iatrogenic laryngotracheal stenosis and idiopathic subglottic stenosis, and co-expressed with CD4 in peritracheal epithelial cells (186). In oral squamous cell carcinoma, the expression of PD-1 and PD-L1 in tumor tissues is significantly increased, and it is further increased when combined with oral mucosal fibrosis, and the level of PD-L1 is positively correlated with the occurrence of fibrosis (187). In PH-associated vascular fibrosis, the expression of PD-L1 is significantly increased and mediated by JAK/STAT1 pathway. Upregulation of PD-L1 can further activate pyroptosis of pulmonary artery smooth muscle cells and promote vascular fibrosis, eventually leading to PH (188). It can be concluded that immune and non-immune regulatory signals mediated by PD-1/PD-L1 pathway are involved in various fibrotic diseases.

## 8 Conclusions

In this review, we summarize the research progress of the PD-1/PD-L1 axis and discuss its potential role in fibrotic diseases. PD-1 and PD-L1 are important immune checkpoint molecules to maintain immune homeostasis, which are widely expressed in immune cells such as T cells, B cells, macrophages, monocytes, DC, and NK cells, and non-immune cells such as epithelial cells, fibroblasts, and endothelial cells. They are distributed in different forms on the cell membrane, cytoplasm, nucleus, and even extracellular, and exert diverse immunomodulatory functions. The expression of PD-1 and PD-L1 can be activated by a variety of pro-oncogene, inflammatory factor, and growth factor signals, which are regulated by transcription factors, kinases, and miRNAs at different levels including transcriptional, post-transcriptional, epigenetic, translational and post-translational. It has been found that PD-1/PD-L1 axis depends on different mechanisms to regulate a variety of important cellular activities such as T cell activation, proliferation, differentiation, cytokine secretion, apoptosis, macrophage phagocytosis, fibroblast migration and invasion, and tissue repair of MSCs. In addition, the dysregulation of PD-1 and PD-L1 expression has been confirmed in autoimmune diseases, cancers, chronic infections, and other chronic diseases. Importantly, immunomodulatory effects mediated by PD-1/PD-L1 pathway participate in a variety of physiological and pathological immune responses and are essential for immune system homeostasis. Given the promising role of PD-1/PD-L1 axis in biomedicine, its potential as a diagnostic biomarker and therapeutic target for various diseases has been greatly explored. The remarkable achievements of immune checkpoint inhibitors targeting the PD-1/PD-L1 pathway in the clinical tumor immunotherapy have attracted great attention to immune checkpoint blockade therapy, and also laid a foundation for its further application in the treatment of other immune-related diseases.

In recent years, preclinical and clinical studies have preliminarily confirmed that PD-1/PD-L1 signaling is involved in the progression of fibrotic diseases in various organs such as the lung, liver, and kidney. The main finding is that the PD-1/PD-L1 axis has a predominant profibrotic effect. Specifically, PD-1/PD-L1 pathway promotes fibrosis progression by inducing key fibrogenic processes such as macrophage polarization, T cell activation, and transdifferentiation of fibroblasts and epithelial cells. Meanwhile, high expression of PD-1 and PD-L1 has been found in fibrotic tissues of patients in clinical investigations. These evidences further emphasize the close relationship between PD-1/PD-L1 pathway and fibrosis response, and provides new ideas for elucidating the immunomodulatory mechanism of fibrotic diseases. On the other hand, this also suggests that PD-1/PD-L1 may serve as diagnostic and prognostic biomarkers and even potential therapeutic targets for fibrotic diseases. In murine models, blockade of the PD-1/PD-L1 pathway by gene knockout or monoclonal antibodies has been shown to alleviate fibrosis, which preliminarily identified this potential. With further exploration of this point, PD-1/PD-L1 inhibitors may be combined with currently approved anti-fibrosis drugs such as pirfenidone and nintedanib in the near future.

To further clarify the role of PD-1/PD-L1 axis in fibrosis and construct new immunotherapy strategies, it is necessary to fully understand the expression, activation, and regulation mechanisms of PD-1/PD-L1 signaling pathway in fibrosis. However, since the relevant research is still in the preliminary stage and the accumulation of evidence is not sufficient, there are still many important scientific issues that need to be further explored. First of all, the pathophysiology of many organ fibrosis types is comparable, yet each type also has its own distinctive features. Therefore, the general immunomodulatory and organ-specific functions of PD-1/PD-L1 signaling in various fibrosis conditions need to be interpreted separately. Secondly, the immunomodulatory effects of PD-1/PD-L1 axis have different performances in different cell types and different immune microenvironments, and fibrosis is a dynamic pathological process with multicellular and multifactorial interactions. Therefore, subsequent studies need to further explore the cell-specific functions of PD-1/PD-L1 signaling in different pathological stages of fibrosis. Thirdly, the initiation factors, regulatory mechanisms, and interactions of PD-1/PD-L1 signaling with other pathways in fibrotic diseases need to be further elucidated. Finally, in addition to PD-1 and PD-L1, inhibitory immune checkpoints such as TIM-3 and LAG3 have also been found to be involved in fibrotic diseases. As immunoregulatory factors with similar functions, whether the synergistic effects of other immune checkpoints should be further clarified when studying PD-1/PD-L1 signaling should be paid attention to.

All in all, PD-1/PD-L1 axis mediates key immunomodulatory and profibrotic signals in fibrosis response, comprehensive exploration and clarification of its specific functions and molecular mechanisms in fibrogenesis are expected to further elucidate the pathogenesis of fibrosis and build promising immunotherapy strategies for fibrotic diseases.

## Author contributions

All authors listed have made a substantial, direct, and intellectual contribution to the work and approved it for publication.
